# Ultralow Auger-Assisted Interlayer Exciton Annihilation in WS_2_/WSe_2_ Moiré Heterobilayers

**DOI:** 10.1021/acs.nanolett.3c04688

**Published:** 2024-01-29

**Authors:** Cheng-Syuan Cai, Wei-Yan Lai, Po-Hsuan Liu, Tzu-Chieh Chou, Ro-Ya Liu, Chih-Ming Lin, Shangjr Gwo, Wei-Ting Hsu

**Affiliations:** † Department of Physics, National Tsing Hua University, Hsinchu 30013, Taiwan; ‡ National Synchrotron Radiation Research Center, Hsinchu 30076, Taiwan; § Research Center for Applied Sciences, Academia Sinica, Taipei 11529, Taiwan

**Keywords:** 2D materials, transition metal dichalcogenides, moiré superlattices, interlayer exciton dynamics, exciton−exciton annihilation, upconversion, intra- and interlayer electron interactions

## Abstract

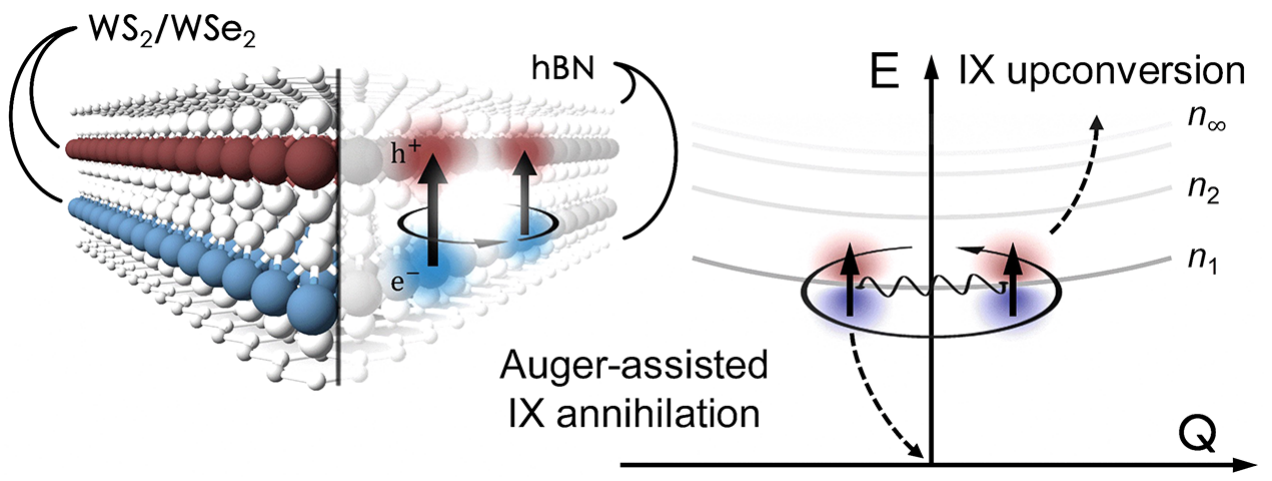

Transition metal dichalcogenide (TMD) heterobilayers have emerged as a promising platform for exploring solid-state quantum simulators and many-body quantum phenomena. Their type II band alignment, combined with the moiré superlattice, inevitably leads to nontrivial exciton interactions and dynamics. Here, we unveil the distinct Auger annihilation processes for delocalized interlayer excitons in WS_2_/WSe_2_ moiré heterobilayers. By fitting the characteristic efficiency droop and bimolecular recombination rate, we quantitatively determine an ultralow Auger coefficient of 1.3 × 10^–5^ cm^2^ s^–1^, which is >100-fold smaller than that of excitons in TMD monolayers. In addition, we reveal selective exciton upconversion into the WSe_2_ layer, which highlights the significance of intralayer electron Coulomb interactions in dictating the microscopic scattering pathways. The distinct Auger processes arising from spatial electron–hole separation have important implications for TMD heterobilayers while endowing interlayer excitons and their strongly correlated states with unique layer degrees of freedom.

Van der Waals (vdW) bilayers composed of transition metal dichalcogenides (TMDs) have emerged as promising candidates for novel excitonic devices^1−7^ and solid-state quantum simulators.^8−12^ Long-lived interlayer excitons (IXs) in vdW bilayers are especially key for applications requiring highly excited active media, such as nanolasers,^13,14^ exciton condensates,^15−17^ and electron–hole plasmas.^18−20^ Beyond these successes, exploring the IX dynamics and interactions related to tunable moiré patterns is essential. In MoSe_2_/WSe_2_ moiré heterobilayers, for example, the exciton–phonon interaction leads to distinct valley dynamics for moiré-trapped IXs and associated charged/spin states.^21−25^ Their microscopic processes thus involve relaxation across moiré potential minima as well as transitions between bright and dark states within the moiré potential. More intriguingly, as the excitation density increases, the transport properties and associated IX phases can change substantially.^19^ In this context, the role of emerging exciton–exciton interactions in moiré superlattices becomes an important subject to explore.^26^

A fundamental process arising from interexcitonic interactions is Auger scattering between two excitons, commonly known as exciton–exciton annihilation (EEA). This process results in the nonradiative recombination (annihilation) of one exciton, with its energy/momentum absorbed by another exciton. Photoluminescence (PL), time-resolved photoluminescence (TRPL), and optical pump–probe spectroscopy have previously been used to study EEA in TMD monolayers, where the PL quantum yield at a high power is greatly reduced due to the hindered exciton population.^27−31^ For monolayer TMD materials, annihilation coefficients in the range of 10^–3^–10^–1^ cm^2^ s^–1^ have been experimentally measured.^32−40^ The microscopic scattering pathways and their intrinsic rate constants have been investigated theoretically.^41,42^ Given the high-lying resonant electronic structures, Auger-mediated upconversion and strain engineering have also become feasible.^43−48^ Notably, recent observations of upconversion electroluminescence and bimolecular recombination in TMD hetero- and homobilayers^20,49^ point to a similar Auger process for IXs. However, systematic investigations of Auger-assisted IX annihilation in TMD moiré heterobilayers remain largely unexplored.

In this work, we demonstrate the distinct Auger-assisted IX annihilation in TMD moiré heterobilayers, which exhibits an ultralow annihilation coefficient and electron-driven selective exciton upconversion. Our samples are hBN-encapsulated WS_2_/WSe_2_ heterobilayers fabricated using standard mechanical exfoliation and stacking procedures (Figure 1a,b). A twist angle (θ) of 58° is measured by polarization-resolved second harmonic generation (see Figure S1). The recombination kinetics of IXs are investigated using PL and TRPL spectroscopy, with Auger-assisted IX annihilation evidenced by the featured efficiency droop and bimolecular population decay. After introducing an IX kinetic model, we quantitatively determine an ultralow Auger coefficient of 1.3 × 10^–5^ cm^2^ s^–1^, which is associated with the weak oscillator strength of IX. Temperature-dependent measurements further validate the model’s accuracy and reveal an increased Auger coefficient at increased temperatures. Finally, layer-selective exciton upconversion is demonstrated by the emergence of prolonged TRPL decays for intralayer excitons in the WSe_2_ layer, highlighting the significance of intralayer electron Coulomb interactions in dictating microscopic scattering pathways.

**Figure 1:**
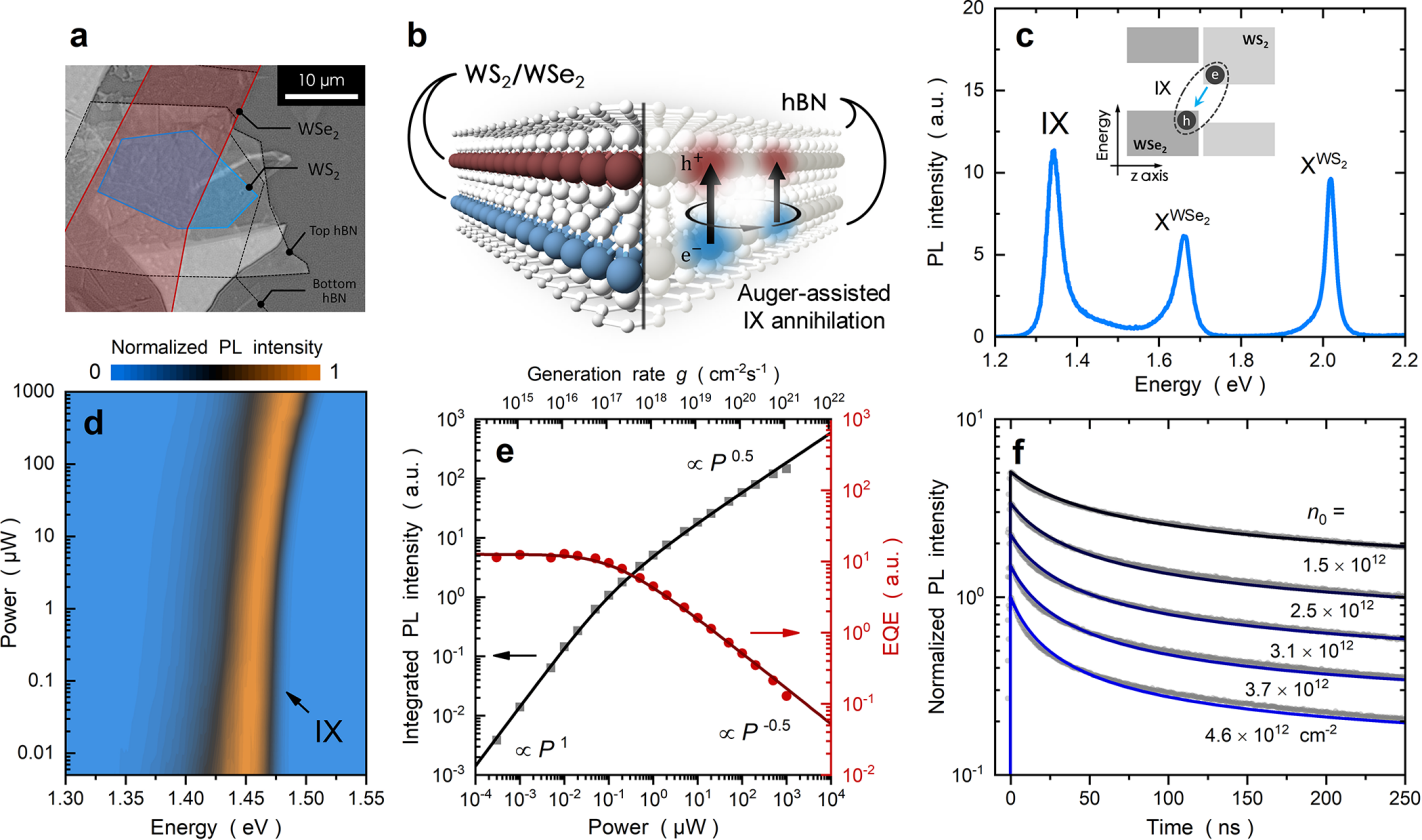
Auger-assisted interlayer exciton annihilation. (a) Optical image and (b) schematic of an hBN-encapsulated WS_2_/WSe_2_ heterobilayer. An IX annihilation event involves the Auger scattering of two IXs, which results in the nonradiative recombination of an IX and the excitation of an energetic IX. (c) Room-temperature PL spectrum revealing the transitions of intralayer excitons X^WS_2_^, X^WSe_2_^, and IXs. The inset shows a schematic of the type II band alignment and low-energy IXs formed by WS_2_ conduction electrons and WSe_2_ valence holes. (d) Normalized power-dependent PL spectra measured at 4 K. With an increasing laser power, the IX peak exhibits an energy blue-shift with an almost constant line width. (e) Integrated PL intensity (*I*_PL_) and EQE as a function of laser power *P* and photocarrier generation rate *g*. *I*_PL_ is obtained by integrating the PL signal over the wavelength range of 800–954 nm (1.30–1.55 eV). (f) TRPL traces measured at various powers (vertically shifted for the sake of clarity). The dots are experimental data, and the curves are fitting results. At high powers, the slope of the integrated PL intensity (EQE) approaches *P*^0.5^ (*P*^–0.5^), while the fast TRPL decay component becomes more prominent. These features demonstrate that the dominant recombination channel is Auger-assisted IX annihilation.

## Interlayer Excitons in WS_2_/WSe_2_ Moiré Heterobilayers

Figure 1c shows a typical room-temperature PL spectrum of the WS_2_/WSe_2_ heterobilayer, displaying signals from intralayer excitons of the WS_2_ (X^WS_2_^) and WSe_2_ (X^WSe_2_^) layers. It also features low-energy PL emission of IXs at 1.35 eV due to the type II band alignment between WS_2_ and WSe_2_ (inset of Figure 1c). The emergence of intralayer moiré excitons, as shown in Figure S1, further confirms the substantial interlayer coupling effect of the heterobilayer.

## Auger-Assisted Interlayer Exciton Annihilation

Conceptually, Auger-assisted IX annihilation is a two-body process (Figure 1b), leading to nonradiative recombination at a rate that depends quadratically on IX density.^27,28^ In this regard, efficient Auger annihilation under high-power excitation often dominates other recombination channels, which the PL can monitor. Figure 1d displays the normalized PL spectra measured at 4 K, showing a continuous increase in PL intensity and peak energy with power.^1−3^ The PL line width, on the other hand, maintains a nearly constant value with power. The recombination mechanism of IXs can be analyzed by the integrated PL intensity (*I*_PL_) and external quantum efficiency (EQE, defined as EQE ∝ *I*_PL/P_) as a function of incident laser power (*P*). As shown in Figure 1e, the slopes of both *I*_PL_ and EQE change roughly around 200 nW, indicating a conversion of the dominant recombination channel. The integrated PL intensity varies from *I*_PL_ ∝ *P*^1.0^ at a low power to *I*_PL_ ∝ *P*^0.5^ at a high power. The EQE, on the other hand, exhibits an efficiency droop with EQE ∝ *P*^–0.5^ at a high power. Because the radiative recombination rate is proportional to IX density *n*_IX_, the data presented above indicate that the dominant recombination channel at a high power is nonradiative recombination with a rate depending on *n*_IX_^2^, a bimolecular recombination process (see Note S1 for rate equation analysis). Thus, the ±0.5 exponents of *I*_PL_ and EQE at high power indicate the Auger-assisted annihilation between two IXs. It is worth noting that the formation of electron–hole plasma at extremely high-density excitations has been ruled out for two reasons. (1) The slope conversion point with an IX density of 8.4 × 10^10^ cm^–2^ (see Note S2) is significantly lower than the Mott transition point reported for TMD heterobilayers,^18,19^ and (2) the characteristic line width broadening effect is absent (see Figure 1d).

To directly visualize the recombination dynamics of IXs, TRPL measurements were performed at 4 K, as shown in Figure 1f. The TRPL trace exhibits multiexponential decay, with the fast decay component becoming more prominent at a high power *P*, which is a typical characteristic of bimolecular population decay. After a preliminary double-exponential analysis (see Figure S2), we find that the intensity ratio of the fast decay component to the slow decay component increases dramatically with *P*. In addition, the time constant of the fast component (τ_1_) decreases with *P*, leaving the time constant of the slow component (τ_2_) nearly unchanged. These features indicate that the fast (slow) component of ∼20 ns (450 ns) largely reflects the Auger-assisted annihilation (radiative recombination) of IXs. The density dependence of the fast decay component further confirms the bimolecular recombination rate, consistent with the power-dependent EQE analysis.

## Modeling Interlayer Exciton Kinetics

**Figure 2:**
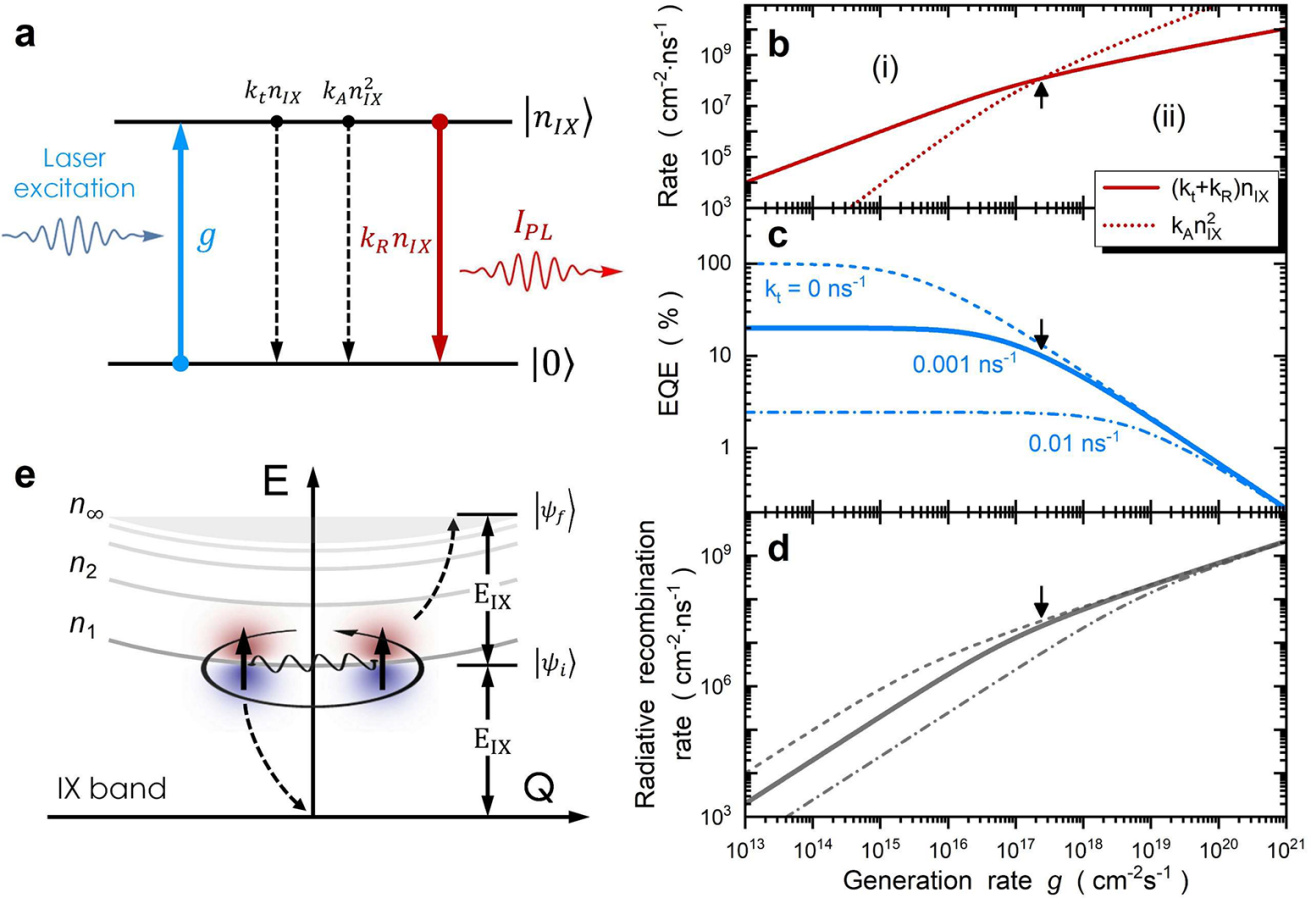
Modeling interlayer exciton kinetics. (a) Schematic of the IX kinetic model, which accounts for the bright IX state with density *n*_IX_. The coefficients for nonradiative recombination, radiative recombination, and Auger-assisted IX annihilation are *k*_t_, *k*_R_, and *k*_A_, respectively. The photocarrier generation rate is given by *g*, and PL intensity *I*_PL_ is equal to *k*_R_*n*__IX__. (b) Simulated recombination rate, (c) EQE, and (d) radiative recombination rate as a function of generation rate. The solid lines in panels c and d are calculated with a *k*_t_ of 0.001 ns^–1^, a *k*_R_ of 2.5 × 10^5^ s^–1^, and a *k*_A_ of 1.3 × 10^–5^ cm^2^ s^–1^. The dashed (dashed–dotted) lines represent simulations with a *k*_t_ of 0 ns^–1^ (0.01 ns^–1^), showing reductions in peak EQE values with *k*_t_. The overall power dependence is governed by competing processes: nonradiative/radiative IX recombination vs Auger-assisted IX annihilation. In this context, the condition (*k*_t_ + *k*_R_)*n*_IX_ = *k*_A_*n*_IX_^2^ (black arrows) divides the power dependence into the low-density regime (i) and the high-density regime (ii). (e) Schematic energy diagram depicting the IX band dispersion. The annihilation of one IX is accompanied by the excitation of another IX into the continuum, where the total energy and momentum are conserved. The microscopic Auger process between IXs can be calculated using Fermi’s golden rule, where the annihilation rate is proportional to the product of the decay rate and the absorption rate of the involved IXs.

A two-level model is considered here to capture the IX dynamics and to determine the rate constants quantitatively. As shown in Figure 2a, the rate equation of the bright IX state (|*n*_IX_⟩) at the K valley can be expressed as
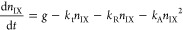
1where *n*_IX_ is the IX density and *g* is the photocarrier generation rate. *k*_t_, *k*_R_, and *k*_A_ are the coefficients for nonradiative recombination, radiative recombination, and Auger-assisted IX annihilation, respectively. To fit the TRPL data, generation rate *g* has been replaced by initial photocarrier density *n*_0_. By assuming a typical set of rate constants, we can model the recombination kinetics and EQE as functions of *g*, as shown in panels b–d of Figure 2. The slope changes of *I*_PL_ and EQE can be reproduced in this simulation, and the effects of temperature (*k*_t_) have also been anticipated. The entire power dependence can be understood by the competition between low-order and high-order processes, i.e., between nonradiative/radiative recombination and Auger-assisted IX annihilation. Separated by the condition (*k*_t_ + *k*_R_)*n*_IX_ = *k*_A_*n*_IX_^2^ (indicated by arrows in Figure 2b–d), the power dependence can be divided into two regimes: (i) the low-density regime dominated by nonradiative/radiative recombination and (ii) the high-density regime dominated by Auger annihilation. In the low-density regime (i), EQE remains nearly constant, while *I*_PL_ exhibits a linear power dependence. In the high-density regime (ii), when *n*_IX_ ≥ (*k*_t_ + *k*_R_)/*k*_A_, the EQE droop occurs because IX annihilation begins to dominate other recombination processes.

As shown by the solid curves in panels e and f of Figure 1, the proposed IX kinetic model provides an excellent fit to the entire set of experimental data, including *I*_PL_, EQE, and TRPL traces. At 4 K, we obtain an ultralow IX annihilation coefficient (*k*_A_) of 1.3 × 10^–5^ cm^2^ s^–1^, which is >2 orders of magnitude lower than that of excitons in monolayer TMDs. The complete fitting parameters and error bars are shown in Figure S3. Microscopically, an Auger-assisted IX annihilation event is directly linked to one IX recombined with its energy being absorbed by another IX to its excited state concurrently (as illustrated in Figure 2e). As a result, the transition rate of the annihilated IX and excited IX is connected with the energy transfer rate during the dipole interaction. According to Fermi’s golden rule,^39^ the IX annihilation rate can be expressed as the product of the decay rate and absorption rate of the involved IXs (see Note S3). In this context, a weak oscillator strength (a long radiative lifetime) leads to a low Auger coefficient and thus an inverse scaling effect.^39^ Given the weaker oscillator strength of IXs compared with excitons in TMD monolayers, it is not surprising that the annihilation coefficient is greatly reduced.

## Influence of Temperature on Interlayer Exciton Kinetics

**Figure 3:**
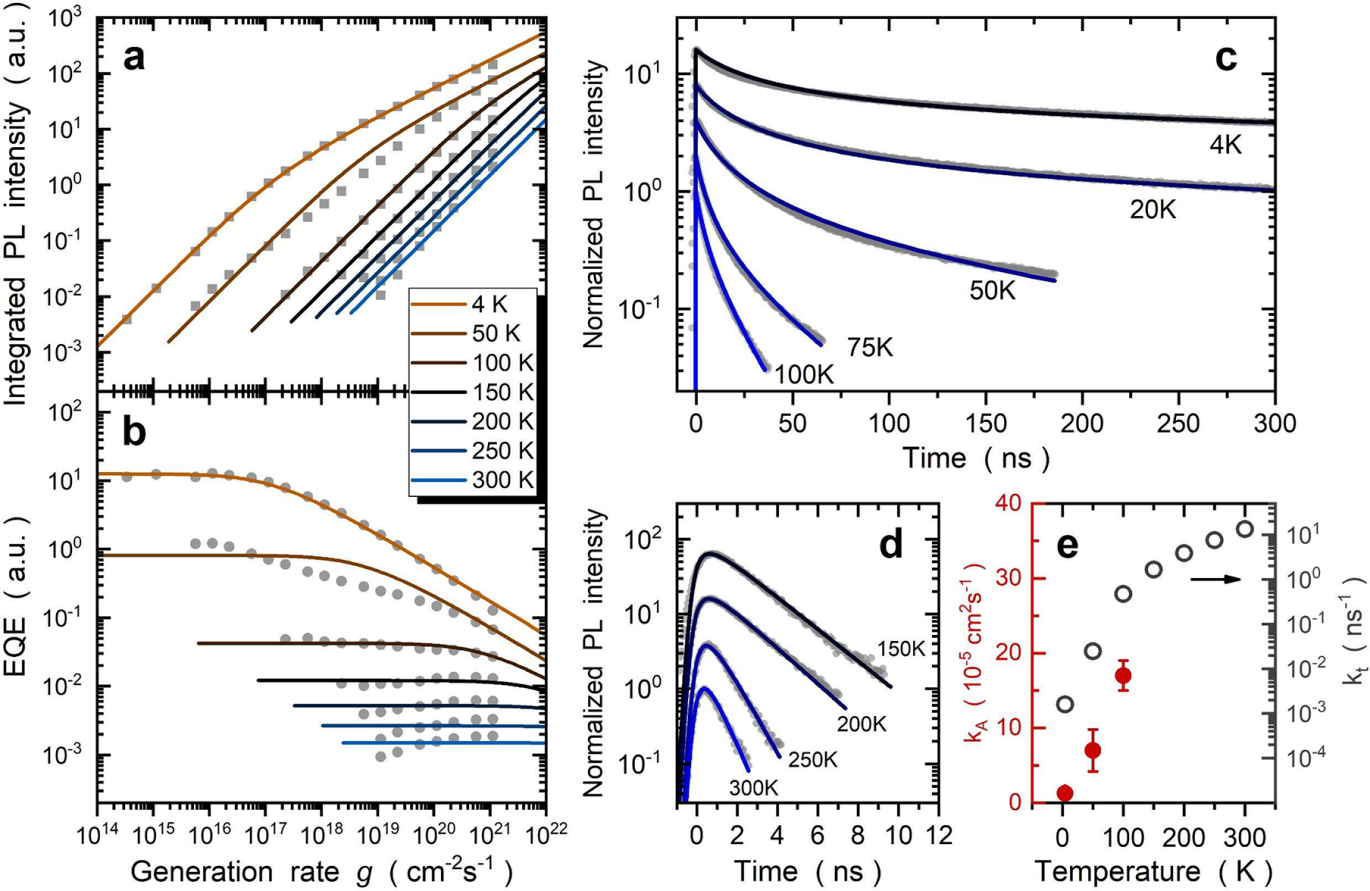
Influence of temperature on interlayer exciton kinetics. (a and b) Power dependence of *I*_PL_ and EQE, respectively, measured at increased temperatures. (c and d) Temperature-dependent TRPL traces vertically shifted for the sake of clarity. The dots are experimental data, and the curves are fitting results. The data in panels a–c are fitted by the kinetic model, while the data in panel d are fitted by single-exponential decay functions (see Figure S4). (e) Experimentally determined *k*_A_ and *k*_t_ values at different temperatures. Auger annihilation coefficient *k*_A_ increases with temperature, while *k*_t_ shows an explicit thermal activation behavior. At high temperatures, thermally activated *k*_t_ significantly decreases *I*_PL_ and peak EQE values, causing the slope conversion point to occur at a higher power. When *T* ≥ 150 K, the rapid TRPL decay component also reflects the dominant role of nonradiative recombination.

Temperature-dependent measurements further validate the accuracy of the IX kinetic model and reveal an increase in the Auger coefficient at increased temperatures. As shown in panels a and b of Figure 3, the considerable decreases in *I*_PL_ and peak EQE values at increased temperatures indicate thermal activation of *k*_t_. The reduction in the IX density further causes the slope conversion point to occur at a higher power. These behaviors are consistent with the kinetic model presented in panels c and d of Figure 2. Meanwhile, the TRPL traces (shown in panels c and d of Figure 3) become much shorter at increased temperatures and eventually become single-exponential decays at ≥150 K, also reflecting the dominant role of nonradiative recombination (see also Figure S4).

As demonstrated by the solid curves in Figure 3a–d, the kinetic model gives an excellent fit to the entire set of experimental data. We find that *k*_A_ can be precisely determined only at temperatures below 150 K; after that, an efficient *k*_t_ dominates the whole IX recombination. Two separate fitting sets are employed here to examine the roles of *k*_A_ and *k*_t_. In the first fitting set, as shown in Note S4, we change only *k*_t_ while keeping the other coefficients fixed. This fitting set already fits all of the experimental data well and captures the effects of thermally activated *k*_t_. In the second fitting set, as shown in Figure 3, we further freed other coefficients to obtain the best fit. As summarized in Figure 3e, IX annihilation coefficient *k*_A_ increases with temperature, while *k*_t_ shows an explicit thermal activation behavior. The significant increase in *k*_t_ at high temperatures is associated with phonon-assisted processes, which may involve trap-assisted recombination, relaxation across moiré potential minima, and transitions between bright and dark states (see Note S5).^22−25^ We further highlight that the increase in *k*_A_ requires a deeper theoretical analysis and may imply the presence of intervalley scattering pathways.^38^ Our results demonstrate that nonradiative recombination must be considered when studying IX kinetics at high temperatures, especially for such a low Auger-assisted IX annihilation rate.

## Signatures of Auger-Mediated Selective Exciton Upconversion

**Figure 4:**
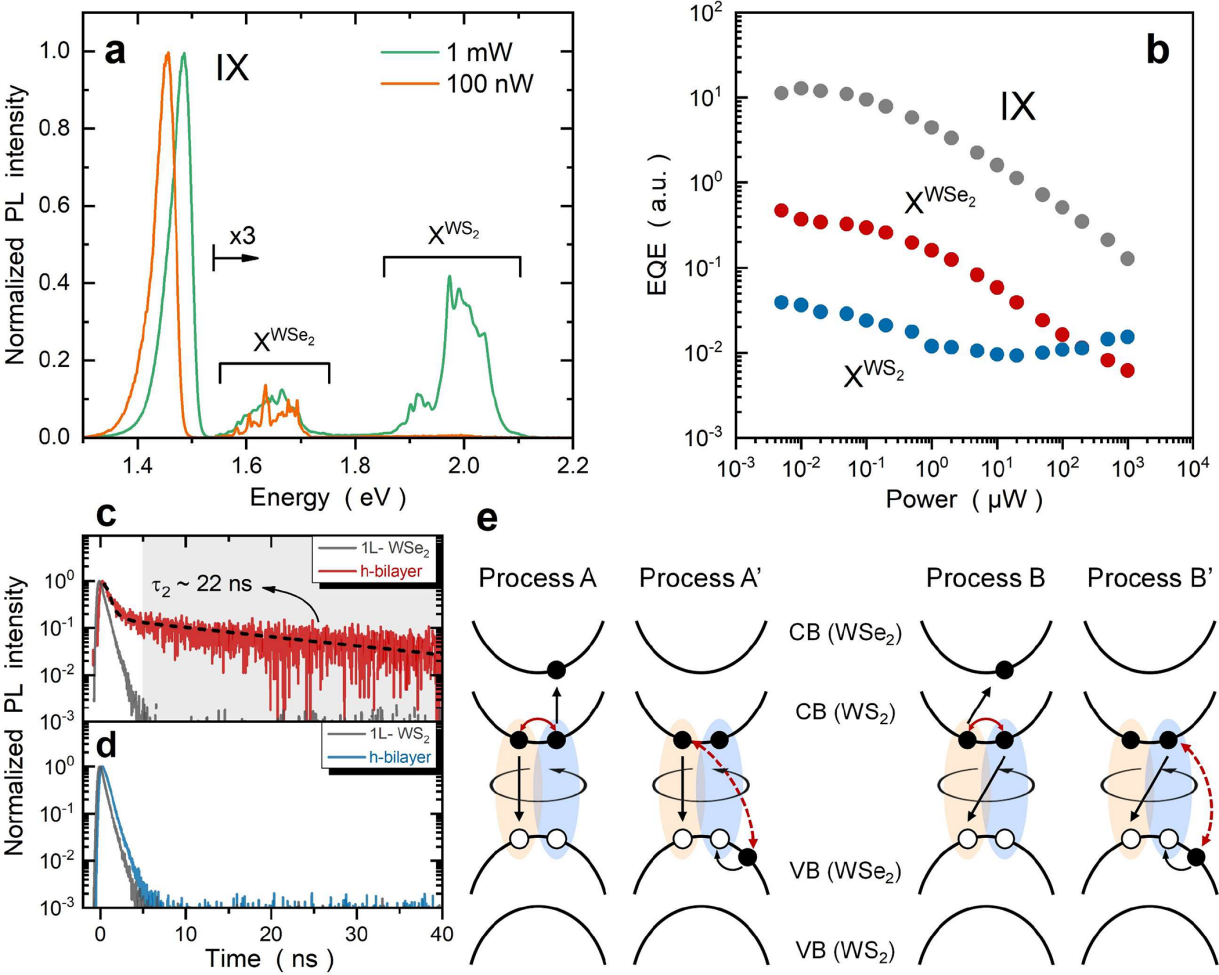
Auger-mediated selective exciton upconversion. (a) Normalized PL spectra show that X^WSe_2_^ (X^WS_2_^) exhibits a power dependence similar (different) to that of IX. Spectra with energies of >1.54 eV have been magnified by a factor of 3 to better visualize intralayer excitons. (b) Power-dependent EQEs show similar efficiency droops for IX and X^WSe_2_^, whereas the X^WS_2_^ efficiency remains roughly unchanged. TRPL traces of (c) WSe_2_ excitons and (d) WS_2_ excitons in monolayer and heterobilayer samples. An emerging decay with a prolonged lifetime (τ_2_) of ≈22 ns (gray region) has been discovered for WSe_2_ excitons in the heterobilayer. Notably, this behavior is not found for WS_2_ excitons. Because τ_2_ is very close to the time constant of IX annihilation (∼20 ns), this observation implies that the emerging decay reflects the regeneration of WSe_2_ excitons via Auger-mediated IX upconversion. The presence (absence) of prolonged decays for WSe_2_ excitons (WS_2_ excitons) further reveals layer-selective exciton upconversion, in which electrons (holes) are allowed (forbidden) to be upconverted into the WSe_2_ (WS_2_) layer. (e) Schematic of the microscopic Auger annihilation pathways,^41,42,54^ which includes an annihilated IX (yellow) and an excited IX (blue). The black arrows represent real transitions. The solid (dashed) red arrows represent electron scatterings via intralayer (interlayer) Coulomb interactions. The process A (A′) represents a scattering event between a conduction electron and a conduction (valence) electron, which can excite an energetic electron (hole) following IX annihilation. Process B (B′) is the exchange counterpart of process A (A′). It should be noted that processes A and B (A′ and B′) are intralayer (interlayer) scattering events. As a result, processes A and B are expected to be more efficient in Auger-assisted IX annihilation, which further results in selective exciton upconversion into the WSe_2_ layer.

The microscopic Auger annihilation mechanism was further investigated by intralayer excitons in the WS_2_ (X^WS_2_^) and WSe_2_ (X^WSe_2_^) layers. Figure 4a shows two PL spectra obtained at very different powers, displaying a similar power dependence for X^WSe_2_^ and IX, whereas that of X^WS_2_^ is somewhat different. Note that the multiple emission lines are typical features of intralayer exciton complexes in tungsten-based TMDs.^50−53^ The analysis of power-dependent EQE is presented in Figure 4b, showing a similar efficiency droop for X^WSe_2_^ and IX, while the efficiency of X^WS_2_^ remains roughly unchanged. However, as shown in Figure S5, excitons in both monolayer WS_2_ and WSe_2_ samples exhibit no EQE droop, indicating that EEA is not the dominant recombination channel in this excitation range. In this context, the smaller X^WSe_2_^ population in a type II heterobilayer should make EEA and EQE droop more unlikely. In Figure 4c, we further compare the TRPL traces of WSe_2_ excitons in monolayer and heterobilayer samples. Surprisingly, an emerging TRPL decay with a prolonged lifetime (τ_2_) of ≈22 ns has been discovered in the WS_2_/WSe_2_ heterobilayer. However, as shown in Figure 4d, this behavior is not found for WS_2_ excitons (see also Note S6 and Table S1).

Instead of direct EEA and interactions between intralayer excitons and IXs (Note S6), our findings point to Auger-mediated exciton upconversion. Because τ_2_ is very close to the time constant of IX annihilation (∼20 ns), this observation implies that the emerging decay reflects the regeneration of WSe_2_ excitons via Auger-mediated IX upconversion, similar to the upconversion effects observed in TMD heterostructures.^49^ Microscopically, an IX annihilation event excites a high-energy IX, which may subsequently be converted into a WSe_2_ exciton. This regenerated WSe_2_ exciton later emits PL on a longer time scale. Given that WSe_2_ excitons recombine extremely fast, the prolonged TRPL decay thus reflects the upconversion rate. Moreover, the presence (absence) of prolonged TRPL decays in the WSe_2_ (WS_2_) layer reveals layer-selective carrier regeneration, in which electrons (holes) are allowed (forbidden) to be upconverted into the WSe_2_ (WS_2_) layer.

The layer-selective exciton upconversion is mainly governed by electron Coulomb interactions,^41,42,54−56^ with intralayer (interlayer) scattering events expected to play a key (minor) role. Microscopic Auger processes have been studied before in TMD monolayers,^41,42,54^ but in TMD heterobilayers, the conduction and valence band edges are located in different layers, which leads to anisotropic scattering. Figure 4e summarizes the microscopic pathways of Auger-assisted IX annihilation in a WS_2_/WSe_2_ heterobilayer. In process A, IX annihilation is achieved by a scattering event between two WS_2_ conduction electrons, which results in the excitation of an energetic electron into the higher conduction band. In process A′, IX annihilation is achieved by a scattering event between a WS_2_ conduction electron and a WSe_2_ valence electron, which results in the excitation of an energetic hole into the deeper valence band. Processes B and B′ denote their corresponding exchange processes. Note that processes A and B (A′ and B′) can lead to exciton upconversion into the WSe_2_ (WS_2_) layer. The critical distinction between these processes is that processes A and B involve an intralayer electron scattering event, whereas processes A′ and B′ rely on an interlayer electron scattering event. As a result, electron-driven exciton upconversion via processes A and B should be considerably more efficient. Most critically, the resulting selective exciton upconversion into the WSe_2_ layer is consistent with our experimental results.

Our findings highlight the significance of intralayer electron Coulomb interactions in dictating the many-body effects in TMD hetero- and homobilayers. We note that further study is required to explore the influence of high-lying resonant bands, intravalley/intervalley scatterings, and interactions between intralayer excitons and IXs.^38,43−48^ Electron tunneling processes can also be significantly affected by hybridized electronic bands with delocalized wave functions and long-range Coulomb interactions.^57^ We underline that upconversion effects facilitated by interexcitonic interactions may play a more critical role in future TMD-based excitonic devices, particularly those containing moiré superlattices. One such possibility is strain engineering of the recombination pathway for IXs in moiré vdW bilayers.^47,48^ In addition, we remark that the distinct IX dynamics found here not only imply a rich moiré exciton phenomena in TMD heterobilayers but also underline the importance of the exciton phase. Specifically, unlike moiré-trapped IXs in MoSe_2_/WSe_2_ heterobilayers, our results show Auger processes of delocalized IXs in WS_2_/WSe_2_ heterobilayers. As discussed in Note S5, spatially resolved PL reveals micrometer-scale IX diffusion, demonstrating that IXs are delocalized from the moiré potential. Our findings, combined with the large IX separation distance, indicate that IX diffusion must precede Auger annihilation, which is consistent with a mobile exciton gas propagating within the moiré superlattice.^58−60^

The IX dynamics in WS_2_/WSe_2_ moiré heterobilayers has been investigated using density-dependent PL spectroscopy. Our experiments firmly establish the Auger-assisted IX annihilation process with an ultralow coefficient of 1.3 × 10^–5^ cm^2^ s^–1^ at low temperatures, where the proposed kinetic model accurately captures the featured efficiency droop and bimolecular recombination rate. The ultralow Auger coefficient is linked to the weak oscillator strength of IX, which is consistent with the inverse scaling effect. Temperature-dependent measurements further reveal an increase in the Auger coefficient at increased temperatures and validate the model’s accuracy, with the low- and high-density regimes being well resolved. Finally, we unveil Auger-mediated selective exciton upconversion, highlighting the importance of the intralayer electron Coulomb interactions. The distinct Auger processes demonstrated here have important implications for TMD moiré heterostructures, encouraging further investigations to exploit quantum yields via strain engineering,^47,48^ Coulomb engineering,^61−65^ and twist angle.^66^ The ultralow Auger coefficient of long-lived IXs makes it easier to sustain under high-level excitation, rendering them as a promising platform for exploring highly correlated quantum phenomena and solid-state quantum simulators. In addition, the selective exciton upconversion offers unique layer degrees of freedom for IXs and their strongly correlated states.

## Materials and Methods

### Sample Preparation

High-quality single-crystal hexagonal BN (hBN), WS_2_, and WSe_2_ (purchased from 2D Semiconductors and HQ Graphene) were employed in the experiments. TMD monolayers were first mechanically exfoliated on polydimethylsiloxane (PDMS) substrates. The hBN-encapsulated WS_2_/WSe_2_ heterobilayers were subsequently assembled in a layer-by-layer manner on SiO_2_/Si substrates using a modified dry transfer technique^67^ in a home-built two-dimensional material transfer system. Finally, the samples were annealed at 150 °C in a vacuum environment with a pressure of 10 mTorr.

### Optical Measurements

All optical characterizations, including PL, TRPL, Raman, and SHG spectroscopies, were performed in a home-built optical microscope. The sample was cooled to 4 K using a cryogen-free low-vibration cryostat equipped with a three-axis piezo-positioner for low-temperature measurements. The excitation source was focused on the sample by a 30× objective lens (NA = 0.35). The optical signal was delivered to a 0.75 m monochromator and detected by a nitrogen-cooled CCD camera. PL and Raman experiments were performed using a 532 nm continuous-wave solid-state laser, while TRPL was carried out using a picosecond pulsed laser diode (532 nm and 2.5 MHz). The entire IX emission was collected using an 800 nm long-pass filter, and TRPL traces were recorded by the time-correlated single-photon counting technique with an overall time resolution of 450 ps (see Figure S4). For SHG measurements, a mode-locked Ti:sapphire laser provided the fundamental field with a pulse width of 150 fs and a repetition rate of 80 MHz. The polarization of the fundamental laser (SHG signal) was selected (analyzed) by individual sets of linear polarizers and half-wave plates.

## Data Availability

All data needed to evaluate the conclusions in the paper are present in the paper and the Supporting Information.

## Supporting Information Available

Additional data and analysis, including intralayer moiré excitons, double-exponential analysis of TRPL decay traces, determination of fitting error bars, TRPL trace fitting at high temperatures, power-dependent PL of monolayer WS_2_ and WSe_2_, power-dependent PL intensity and EQE, estimation of photoexcited IX density, calculation of the IX annihilation rate using Fermi’s golden rule, fitting of temperature-dependent IX kinetics, Auger recombination of delocalized IXs, and negligible effect of intralayer excitons on IX recombination (PDF)

## References

[ref1] Rivera P.; Schaibley J. R.; Jones A. M.; Ross J. S.; Wu S.; Aivazian G.; Klement P.; Seyler K.; Clark G.; Ghimire N. J.; Yan J.; Mandrus D. G.; Yao W.; Xu X. Observation of long-lived interlayer excitons in monolayer MoSe_2_-WSe_2_ heterostructures. Nat. Commun. 2015, 6, 6242 10.1038/ncomms7242. 25708612

[ref2] Rivera P.; Yu H.; Seyler K. L.; Wilson N. P.; Yao W.; Xu X. Interlayer valley excitons in heterobilayers of transition metal dichalcogenides. Nat. Nanotechnol. 2018, 13, 1004–1015. 10.1038/s41565-018-0193-0. 30104622

[ref3] Jiang Y.; Chen S.; Zheng W.; Zheng B.; Pan A. Interlayer exciton formation, relaxation, and transport in TMD van der Waals heterostructures. Light: Sci. Appl. 2021, 10, 72 10.1038/s41377-021-00500-1. 33811214 PMC8018964

[ref4] Unuchek D.; Ciarrocchi A.; Avsar A.; Watanabe K.; Taniguchi T.; Kis A. Room-temperature electrical control of exciton flux in a van der Waals heterostructure. Nature 2018, 560, 340–344. 10.1038/s41586-018-0357-y. 30046107

[ref5] Ciarrocchi A.; Unuchek D.; Avsar A.; Watanabe K.; Taniguchi T.; Kis A. Polarization switching and electrical control of interlayer excitons in two-dimensional van der Waals heterostructures. Nat. Photonics 2019, 13, 131–136. 10.1038/s41566-018-0325-y. 30886643 PMC6420072

[ref6] Jauregui L. A.; Joe A. Y.; Pistunova K.; Wild D. S.; High A. A.; Zhou Y.; Scuri G.; De Greve K.; Sushko A.; Yu C.-H.; Taniguchi T.; Watanabe K.; Needleman D. J.; Lukin M. D.; Park H.; Kim P. Electrical control of interlayer exciton dynamics in atomically thin heterostructures. Science 2019, 366, 870–875. 10.1126/science.aaw4194. 31727834

[ref7] Unuchek D.; Ciarrocchi A.; Avsar A.; Sun Z.; Watanabe K.; Taniguchi T.; Kis A. Valley-polarized exciton currents in a van der Waals heterostructure. Nat. Nanotechnol. 2019, 14, 1104–1109. 10.1038/s41565-019-0559-y. 31636411 PMC6897556

[ref8] Seyler K. L.; Rivera P.; Yu H.; Wilson N. P.; Ray E. L.; Mandrus D. G.; Yan J.; Yao W.; Xu X. Signatures of moiré-trapped valley excitons in MoSe_2_/WSe_2_ heterobilayers. Nature 2019, 567, 66–70. 10.1038/s41586-019-0957-1. 30804526

[ref9] Tran K.; Moody G.; Wu F.; Lu X.; Choi J.; Kim K.; Rai A.; Sanchez D. A.; Quan J.; Singh A.; Embley J.; Zepeda A.; Campbell M.; Autry T.; Taniguchi T.; Watanabe K.; Lu N.; Banerjee S. K.; Silverman K. L.; Kim S.; Tutuc E.; Yang L.; MacDonald A. H.; Li X. Evidence for moiré excitons in van der Waals heterostructures. Nature 2019, 567, 71–75. 10.1038/s41586-019-0975-z. 30804527 PMC11493145

[ref10] Jin C.; Regan E. C.; Yan A.; Iqbal Bakti Utama M.; Wang D.; Zhao S.; Qin Y.; Yang S.; Zheng Z.; Shi S.; Watanabe K.; Taniguchi T.; Tongay S.; Zettl A.; Wang F. Observation of moiré excitons in WSe_2_/WS_2_ heterostructure superlattices. Nature 2019, 567, 76–80. 10.1038/s41586-019-0976-y. 30804525

[ref11] Alexeev E. M.; Ruiz-Tijerina D. A.; Danovich M.; Hamer M. J.; Terry D. J.; Nayak P. K.; Ahn S.; Pak S.; Lee J.; Sohn J. I.; Molas M. R.; Koperski M.; Watanabe K.; Taniguchi T.; Novoselov K. S.; Gorbachev R. V.; Shin H. S.; Fal’ko V. I.; Tartakovskii A. I. Resonantly hybridized excitons in moiré superlattices in van der Waals heterostructures. Nature 2019, 567, 81–86. 10.1038/s41586-019-0986-9. 30842637

[ref12] Kennes D. M.; Claassen M.; Xian L.; Georges A.; Millis A. J.; Hone J.; Dean C. R.; Basov D. N.; Pasupathy A. N.; Rubio A. Moiré heterostructures as a condensed-matter quantum simulator. Nat. Phys. 2021, 17, 155–163. 10.1038/s41567-020-01154-3.

[ref13] Paik E. Y.; Zhang L.; Burg G. W.; Gogna R.; Tutuc E.; Deng H. Interlayer exciton laser of extended spatial coherence in atomically thin heterostructures. Nature 2019, 576, 80–84. 10.1038/s41586-019-1779-x. 31768043

[ref14] Liu Y.; Fang H.; Rasmita A.; Zhou Y.; Li J.; Yu T.; Xiong Q.; Zheludev N.; Liu J.; Gao W. Room temperature nanocavity laser with interlayer excitons in 2D heterostructures. Sci. Adv. 2019, 5, eaav4506 10.1126/sciadv.aav4506. 31032409 PMC6486267

[ref15] Fogler M. M.; Butov L. V.; Novoselov K. S. High-temperature superfluidity with indirect excitons in van der Waals heterostructures. Nat. Commun. 2014, 5, 4555 10.1038/ncomms5555. 25065343

[ref16] Wu F.-C.; Xue F.; MacDonald A. H. Theory of two-dimensional spatially indirect equilibrium exciton condensates. Phys. Rev. B 2015, 92, 165121 10.1103/PhysRevB.92.165121.

[ref17] Wang Z.; Rhodes D. A.; Watanabe K.; Taniguchi T.; Hone J. C.; Shan J.; Mak K. F. Evidence of high-temperature exciton condensation in two-dimensional atomic double layers. Nature 2019, 574, 76–80. 10.1038/s41586-019-1591-7. 31578483

[ref18] Wang J.; Ardelean J.; Bai Y.; Steinhoff A.; Florian M.; Jahnke F.; Xu X.; Kira M.; Hone J.; Zhu X.-Y. Optical generation of high carrier densities in 2D semiconductor heterobilayers. Sci. Adv. 2019, 5, eaax0145 10.1126/sciadv.aax0145. 31548986 PMC6744266

[ref19] Wang J.; Shi Q.; Shih E. M.; Zhou L.; Wu W.; Bai Y.; Rhodes D.; Barmak K.; Hone J.; Dean C. R.; Zhu X.-Y. Diffusivity reveals three distinct phases of interlayer excitons in MoSe_2_/WSe_2_ heterobilayers. Phys. Rev. Lett. 2021, 126, 106804 10.1103/PhysRevLett.126.106804. 33784140

[ref20] Siday T.; Sandner F.; Brem S.; Zizlsperger M.; Perea-Causin R.; Schiegl F.; Nerreter S.; Plankl M.; Merkl P.; Mooshammer F.; Huber M. A.; Malic E.; Huber R. Ultrafast nanoscopy of high-density exciton phases in WSe_2_. Nano Lett. 2022, 22, 2561–2568. 10.1021/acs.nanolett.1c04741. 35157466

[ref21] Brotons-Gisbert M.; Baek H.; Campbell A.; Watanabe K.; Taniguchi T.; Gerardot B. D. Moiré-trapped interlayer trions in a charge-tunable WSe_2_/MoSe_2_ heterobilayer. Phys. Rev. X 2021, 11, 031033 10.1103/PhysRevX.11.031033.

[ref22] Shinokita K.; Miyauchi Y.; Watanabe K.; Taniguchi T.; Matsuda K. Resonant coupling of a moiré exciton to a phonon in a WSe_2_/MoSe_2_ heterobilayer. Nano Lett. 2021, 21, 5938–5944. 10.1021/acs.nanolett.1c00733. 34269588

[ref23] Shinokita K.; Watanabe K.; Taniguchi T.; Matsuda K. Valley relaxation of the moiré excitons in a WSe_2_/MoSe_2_ heterobilayer. ACS Nano 2022, 16, 16862–16868. 10.1021/acsnano.2c06813. 36169188

[ref24] Kim H.; Dong D.; Okamura Y.; Shinokita K.; Watanabe K.; Taniguchi T.; Matsuda K. Dynamics of moiré trion and its valley polarization in a microfabricated WSe_2_/MoSe_2_ heterobilayer. ACS Nano 2023, 17, 13715–13723. 10.1021/acsnano.3c02952. 37450661

[ref25] Kim H.; Aino K.; Shinokita K.; Zhang W.; Watanabe K.; Taniguchi T.; Matsuda K. Dynamics of moiré exciton in a twisted MoSe_2_/WSe_2_ heterobilayer. Adv. Opt. Mater. 2023, 11, 2300146 10.1002/adom.202300146.

[ref26] Brem S.; Malic E. Bosonic delocalization of dipolar moiré excitons. Nano Lett. 2023, 23, 4627–4633. 10.1021/acs.nanolett.3c01160. 37184441

[ref27] Sun D.; Rao Y.; Reider G. A.; Chen G.; You Y.; Brézin L.; Harutyunyan A. R.; Heinz T. F. Observation of rapid exciton-exciton annihilation in monolayer molybdenum disulfide. Nano Lett. 2014, 14, 5625–5629. 10.1021/nl5021975. 25171389

[ref28] Kumar N.; Cui Q.; Ceballos F.; He D.; Wang Y.; Zhao H. Exciton-exciton annihilation in MoSe_2_ monolayers. Phys. Rev. B 2014, 89, 125427 10.1103/PhysRevB.89.125427.

[ref29] Salehzadeh O.; Tran N. H.; Liu X.; Shih I.; Mi Z. Exciton kinetics, quantum efficiency, and efficiency droop of monolayer MoS_2_ light-emitting devices. Nano Lett. 2014, 14, 4125–4130. 10.1021/nl5017283. 24905765

[ref30] Amani M.; Lien D.-H.; Kiriya D.; Xiao J.; Azcatl A.; Noh J.; Madhvapathy S. R.; Addou R.; KC S.; Dubey M.; Cho K.; Wallace R. M.; Lee S.-C.; He J.-H.; Ager J. W. 3rd; Zhang X.; Yablonovitch E.; Javey A. Near-unity photoluminescence quantum yield in MoS_2_. Science 2015, 350, 1065–1068. 10.1126/science.aad2114. 26612948

[ref31] Amani M.; Taheri P.; Addou R.; Ahn G. H.; Kiriya D.; Lien D.-H.; Ager J. W. 3rd; Wallace R. M.; Javey A. Recombination kinetics and effects of superacid treatment in sulfur- and selenium-based transition metal dichalcogenides. Nano Lett. 2016, 16, 2786–2791. 10.1021/acs.nanolett.6b00536. 26978038

[ref32] Mouri S.; Miyauchi Y.; Toh M.; Zhao W.; Eda G.; Matsuda K. Nonlinear photoluminescence in atomically thin layered WSe_2_ arising from diffusion-assisted exciton-exciton annihilation. Phys. Rev. B 2014, 90, 155449 10.1103/PhysRevB.90.155449.

[ref33] Yuan L.; Huang L. Exciton dynamics and annihilation in WS_2_ 2D semiconductors. Nanoscale 2015, 7, 7402–7408. 10.1039/C5NR00383K. 25826397

[ref34] Poellmann C.; Steinleitner P.; Leierseder U.; Nagler P.; Plechinger G.; Porer M.; Bratschitsch R.; Schüller C.; Korn T.; Huber R. Resonant internal quantum transitions and femtosecond radiative decay of excitons in monolayer WSe_2_. Nat. Mater. 2015, 14, 889–893. 10.1038/nmat4356. 26168345

[ref35] Perea-Causin R.; Brem S.; Rosati R.; Jago R.; Kulig M.; Ziegler J. D.; Zipfel J.; Chernikov A.; Malic E. Exciton propagation and halo formation in two-dimensional materials. Nano Lett. 2019, 19, 7317–7323. 10.1021/acs.nanolett.9b02948. 31532993

[ref36] Cordovilla Leon D. F.; Li Z.; Jang S. W.; Deotare P. B. Hot exciton transport in WSe_2_ monolayers. Phys. Rev. B 2019, 100, 241401 10.1103/PhysRevB.100.241401.

[ref37] Zipfel J.; Kulig M.; Perea-Causín R.; Brem S.; Ziegler J. D.; Rosati R.; Taniguchi T.; Watanabe K.; Glazov M. M.; Malic E.; Chernikov A. Exciton diffusion in monolayer semiconductors with suppressed disorder. Phys. Rev. B 2020, 101, 115430 10.1103/PhysRevB.101.115430.

[ref38] Erkensten D.; Brem S.; Wagner K.; Gillen R.; Perea-Causín R.; Ziegler J. D.; Taniguchi T.; Watanabe K.; Maultzsch J.; Chernikov A.; Malic E. Dark exciton-exciton annihilation in monolayer WSe_2_. Phys. Rev. B 2021, 104, L241406 10.1103/PhysRevB.104.L241406.

[ref39] Uddin S. Z.; Rabani E.; Javey A. Universal inverse scaling of exciton-exciton annihilation coefficient with exciton lifetime. Nano Lett. 2021, 21, 424–429. 10.1021/acs.nanolett.0c03820. 33320011

[ref40] Chatterjee S.; Gupta G.; Das S.; Watanabe K.; Taniguchi T.; Majumdar K. Trion-trion annihilation in monolayer WS_2_. Phys. Rev. B 2022, 105, L121409 10.1103/PhysRevB.105.L121409.

[ref41] Konabe S.; Okada S. Effect of Coulomb interactions on optical properties of monolayer transition-metal dichalcogenides. Phys. Rev. B 2014, 90, 155304 10.1103/PhysRevB.90.155304.

[ref42] Steinhoff A.; Jahnke F.; Florian M. Microscopic theory of exciton-exciton annihilation in two-dimensional semiconductors. Phys. Rev. B 2021, 104, 155416 10.1103/PhysRevB.104.155416.

[ref43] Manca M.; Glazov M. M.; Robert C.; Cadiz F.; Taniguchi T.; Watanabe K.; Courtade E.; Amand T.; Renucci P.; Marie X.; Wang G.; Urbaszek B. Enabling valley selective exciton scattering in monolayer WSe_2_ through upconversion. Nat. Commun. 2017, 8, 14927 10.1038/ncomms14927. 28367962 PMC5382264

[ref44] Han B.; Robert C.; Courtade E.; Manca M.; Shree S.; Amand T.; Renucci P.; Taniguchi T.; Watanabe K.; Marie X.; Golub L. E.; Glazov M. M.; Urbaszek B. Exciton states in monolayer MoSe_2_ and MoTe_2_ probed by upconversion spectroscopy. Phys. Rev. X 2018, 8, 031073 10.1103/PhysRevX.8.031073.

[ref45] Linardy E.; Yadav D.; Vella D.; Verzhbitskiy I. A.; Watanabe K.; Taniguchi T.; Pauly F.; Trushin M.; Eda G. Harnessing exciton-exciton annihilation in two-dimensional semiconductors. Nano Lett. 2020, 20, 1647–1653. 10.1021/acs.nanolett.9b04756. 32078334

[ref46] Lin K.-Q.; Ong C. S.; Bange S.; Faria Junior P. E.; Peng B.; Ziegler J. D.; Zipfel J.; Bäuml C.; Paradiso N.; Watanabe K.; Taniguchi T.; Strunk C.; Monserrat B.; Fabian J.; Chernikov A.; Qiu D. Y.; Louie S. G.; Lupton J. M. Narrow-band high-lying excitons with negative-mass electrons in monolayer WSe_2_. Nat. Commun. 2021, 12, 5500 10.1038/s41467-021-25499-2. 34535654 PMC8448890

[ref47] Kim H.; Uddin S. Z.; Higashitarumizu N.; Rabani E.; Javey A. Inhibited nonradiative decay at all exciton densities in monolayer semiconductors. Science 2021, 373, 448–452. 10.1126/science.abi9193. 34437119

[ref48] Uddin S. Z.; Higashitarumizu N.; Kim H.; Rabani E.; Javey A. Engineering exciton recombination pathways in bilayer WSe_2_ for bright luminescence. ACS Nano 2022, 16, 1339–1345. 10.1021/acsnano.1c09255. 35014783

[ref49] Binder J.; Howarth J.; Withers F.; Molas M. R.; Taniguchi T.; Watanabe K.; Faugeras C.; Wysmolek A.; Danovich M.; Fal’ko V. I.; Geim A. K.; Novoselov K. S.; Potemski M.; Kozikov A. Upconverted electroluminescence via Auger scattering of interlayer excitons in van der Waals heterostructures. Nat. Commun. 2019, 10, 2335 10.1038/s41467-019-10323-9. 31133651 PMC6536535

[ref50] Paur M.; Molina-Mendoza A. J.; Bratschitsch R.; Watanabe K.; Taniguchi T.; Mueller T. Electroluminescence from multi-particle exciton complexes in transition metal dichalcogenide semiconductors. Nat. Commun. 2019, 10, 1709 10.1038/s41467-019-09781-y. 30979893 PMC6461636

[ref51] Li Z.; Wang T.; Jin C.; Lu Z.; Lian Z.; Meng Y.; Blei M.; Gao S.; Taniguchi T.; Watanabe K.; Ren T.; Tongay S.; Yang L.; Smirnov D.; Cao T.; Shi S. F. Emerging photoluminescence from the dark-exciton phonon replica in monolayer WSe_2_. Nat. Commun. 2019, 10, 2469 10.1038/s41467-019-10477-6. 31171789 PMC6554274

[ref52] Tang Y.; Mak K. F.; Shan J. Long valley lifetime of dark excitons in single-layer WSe_2_. Nat. Commun. 2019, 10, 4047 10.1038/s41467-019-12129-1. 31492874 PMC6731252

[ref53] He M.; Rivera P.; Van Tuan D.; Wilson N. P.; Yang M.; Taniguchi T.; Watanabe K.; Yan J.; Mandrus D. G.; Yu H.; Dery H.; Yao W.; Xu X. Valley phonons and exciton complexes in a monolayer semiconductor. Nat. Commun. 2020, 11, 618 10.1038/s41467-020-14472-0. 32001715 PMC6992782

[ref54] Wang F.; Wu Y.; Hybertsen M. S.; Heinz T. F. Auger recombination of excitons in one-dimensional systems. Phys. Rev. B 2006, 73, 245424 10.1103/PhysRevB.73.245424.

[ref55] Wang H.; Strait J. H.; Zhang C.; Chan W.; Manolatou C.; Tiwari S.; Rana F. Fast exciton annihilation by capture of electrons or holes by defects via Auger scattering in monolayer metal dichalcogenides. Phys. Rev. B 2015, 91, 165411 10.1103/PhysRevB.91.165411.

[ref56] Moody G.; Schaibley J.; Xu X. Exciton dynamics in monolayer transition metal dichalcogenides. J. Opt. Soc. Am. B 2016, 33, C39–C49. 10.1364/JOSAB.33.000C39. 28890600 PMC5590662

[ref57] Jin C.; Ma E. Y.; Karni O.; Regan E. C.; Wang F.; Heinz T. F. Ultrafast dynamics in van der Waals heterostructures. Nat. Nanotechnol. 2018, 13, 994–1003. 10.1038/s41565-018-0298-5. 30397296

[ref58] Brem S.; Linderälv C.; Erhart P.; Malic E. Tunable phases of moiré excitons in van der Waals heterostructures. Nano Lett. 2020, 20, 8534–8540. 10.1021/acs.nanolett.0c03019. 32970445 PMC7729935

[ref59] Choi J.; Hsu W.-T.; Lu L.-S.; Sun L.; Cheng H.-Y.; Lee M.-H.; Quan J.; Tran K.; Wang C.-Y.; Staab M.; Jones K.; Taniguchi T.; Watanabe K.; Chu M.-W.; Gwo S.; Kim S.; Shih C.-K.; Li X.; Chang W.-H. Moiré potential impedes interlayer exciton diffusion in van der Waals heterostructures. Sci. Adv. 2020, 6, eaba8866 10.1126/sciadv.aba8866. 32967823 PMC7531884

[ref60] Knorr W.; Brem S.; Meneghini G.; Malic E. Exciton transport in a moiré potential: From hopping to dispersive regime. Phys. Rev. Mater. 2022, 6, 124002 10.1103/PhysRevMaterials.6.124002.

[ref61] Hoshi Y.; Kuroda T.; Okada M.; Moriya R.; Masubuchi S.; Watanabe K.; Taniguchi T.; Kitaura R.; Machida T. Suppression of exciton-exciton annihilation in tungsten disulfide monolayers encapsulated by hexagonal boron nitrides. Phys. Rev. B 2017, 95, 241403 10.1103/PhysRevB.95.241403.

[ref62] Lee Y.; Tran T. T.; Kim Y.; Roy S.; Taniguchi T.; Watanabe K.; Jang J. I.; Kim J. Enhanced radiative exciton recombination in monolayer WS_2_ on the hBN substrate competing with nonradiative exciton-exciton annihilation. ACS Photonics 2022, 9, 873–879. 10.1021/acsphotonics.1c01584.

[ref63] Goodman A. J.; Lien D.-H.; Ahn G. H.; Spiegel L. L.; Amani M.; Willard A. P.; Javey A.; Tisdale W. A. Substrate-dependent exciton diffusion and annihilation in chemically treated MoS_2_ and WS_2_. J. Phys. Chem. C 2020, 124, 12175–12184. 10.1021/acs.jpcc.0c04000.

[ref64] Lee Y.; Forte J. D. S.; Chaves A.; Kumar A.; Tran T. T.; Kim Y.; Roy S.; Taniguchi T.; Watanabe K.; Chernikov A.; Jang J. I.; Low T.; Kim J. Boosting quantum yields in two-dimensional semiconductors via proximal metal plates. Nat. Commun. 2021, 12, 7095 10.1038/s41467-021-27418-x. 34876573 PMC8651657

[ref65] Yuan L.; Jeong J.; Chi Kwock K. W.; Yanev E. S.; Grandel M.; Rhodes D. A.; Luk T. S.; Schuck P. J.; Yarotski D.; Hone J. C.; Brener I.; Prasankumar R. P. Manipulation of exciton dynamics in single-layer WSe_2_ using a toroidal dielectric metasurface. Nano Lett. 2021, 21, 9930–9938. 10.1021/acs.nanolett.1c03189. 34797671

[ref66] Yuan L.; Zheng B.; Kunstmann J.; Brumme T.; Kuc A. B.; Ma C.; Deng S.; Blach D.; Pan A.; Huang L. Twist-angle-dependent interlayer exciton diffusion in WS_2_-WSe_2_ heterobilayers. Nat. Mater. 2020, 19, 617–623. 10.1038/s41563-020-0670-3. 32393806

[ref67] Kim K.; Yankowitz M.; Fallahazad B.; Kang S.; Movva H. C.; Huang S.; Larentis S.; Corbet C. M.; Taniguchi T.; Watanabe K.; Banerjee S. K.; LeRoy B. J.; Tutuc E. Van der Waals heterostructures with high accuracy rotational alignment. Nano Lett. 2016, 16, 1989–1995. 10.1021/acs.nanolett.5b05263. 26859527

