# Supplementary material for: Ultralow Auger-Assisted Interlayer Exciton Annihilation in WS2/WSe2 Moiré Heterobilayers
Source: Nano Lett. 2024 Jan 29;24(9):2773–81. doi: 10.1021/acs.nanolett.3c04688 (PMC10921466; doi:10.1021/acs.nanolett.3c04688)
Supplement: Supplementary file 1 — nl3c04688_si_001.pdf [file nl3c04688_si_001.pdf]

Supporting Information for

Ultralow Auger-Assisted Interlayer Exciton Annihilation in WS_2/WSe_2 Moiré Heterobilayers

Authors: Cheng-Syuan Cai,[1,2] Wei-Yan Lai,[1] Po-Hsuan Liu,[1] Tzu-Chieh Chou,[1,2] Ro-Ya Liu,[2] Chih-Ming Lin,[1] Shangjr Gwo,[1] and Wei-Ting Hsu[1,2,3*]

Affiliations:

[1] Department of Physics, National Tsing Hua University, Hsinchu 30013, Taiwan

[2] National Synchrotron Radiation Research Center, Hsinchu 30076, Taiwan

[3] Research Center for Applied Sciences, Academia Sinica, Taipei 11529, Taiwan

[+]Correspondence should be addressed to: wthsu@phys.nthu.edu.tw (W.-T.H.)

This PDF file includes:

Figure S1. Intralayer moiré excitons in WS_2/WSe_2 heterobilayer.

Figure S2. Double-exponential analysis of TRPL decay traces.

Figure S3. Determination of fitting error bars.

Figure S4. TRPL trace fitting at high temperatures.

Figure S5. Power-dependent PL of monolayer WS_2 and WSe_2.

Note S1. The power-dependent PL intensity and EQE.

Note S2. Estimation of photoexcited IX density.

Note S3. Calculation of IX annihilation rate using Fermi's golden rule.

Note S4. Fitting of temperature-dependent IX kinetics.

Note S5. Auger recombination of delocalized IXs in WS_2/WSe_2 heterobilayer.

Note S6. The negligible effect of intralayer excitons on IX recombination.

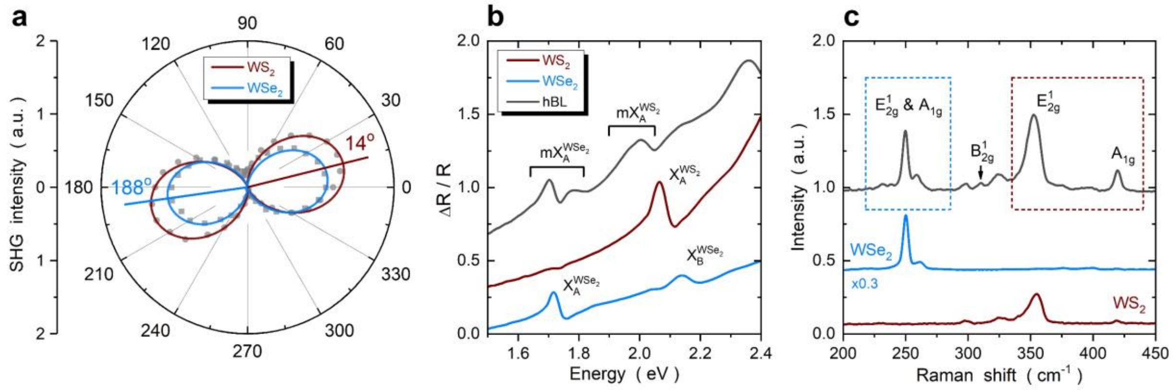

Figure S1. Intralayer moiré excitons in WS_2/WSe_2 heterobilayer. (**a**) A polar plot depicts the polarization-resolved SHG intensity of monolayer WS_2 and WSe_2. The dots are experimental data, and the curves are fitting results. In the experiment, the laser polarization and 2D crystals are left unaltered while the polarization angle of SHG is analyzed. Given that the angle between the crystal armchair direction and the SH field is two times the angle between the crystal armchair direction and the laser field, the twist angle θ can thus be determined using the difference in polarization angle of the SHG signal between monolayers. The twist angle in this WS_2/WSe_2 heterobilayer is found to be $\theta = 58°$. (**b**) Reflection contrast spectra of WS_2 monolayer, WSe_2 monolayer, and WS_2/WSe_2 heterobilayer (hBL). The intralayer moiré excitons $\mathrm{mX}_\mathrm{A}^{\mathrm{WS_2}}$ ($\mathrm{mX}_\mathrm{A}^{\mathrm{WSe_2}}$) possess energies of 1.67-1.80 eV (1.92-2.04 eV), as previously reported [1]. The emergence of multiple peaks demonstrates the formation of moiré minibands. (**c**) Raman characterization of the WS_2/WSe_2 heterobilayer, displaying individual features of the WS_2 and WSe_2 layers. Spectra have been shifted vertically for clarity. The presence of intralayer moiré excitons and interlayer phonon mode B_{2g}^1 demonstrates the high quality of the vdW interface.

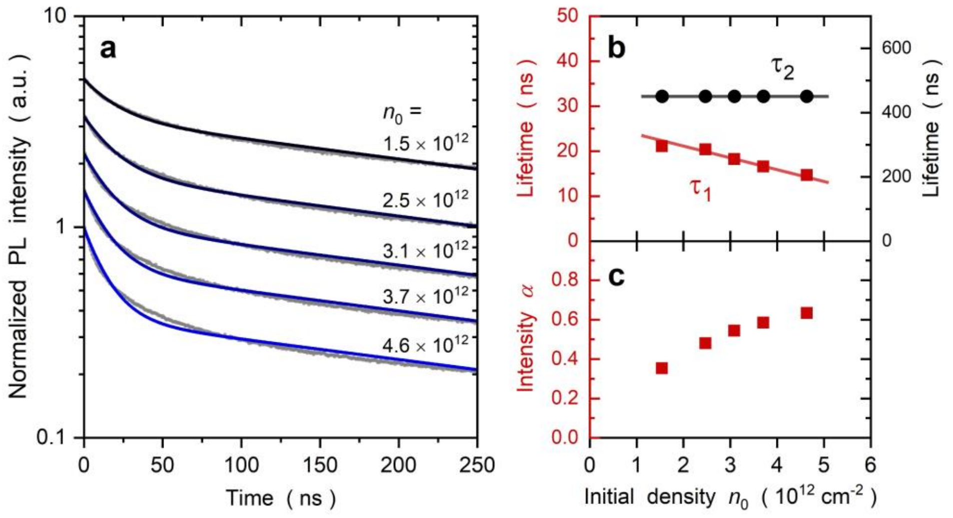

Figure S2. Double-exponential analysis of TRPL decay traces. (**a**) TRPL traces fitted with double-exponential functions. The dots are experimental data, and the curves are fitting results. (**b**) Power-dependent decay time constants τ_1 and τ_2. (**c**) The relative intensity α at various excitation powers. In **Fig. S2a**, we present fits to the TRPL data using a double-exponential decay function: $y(t) = \alpha e^{-t/\tau_1} + (1-\alpha)e^{-t/\tau_2}$, where α and $(1-\alpha)$ denote the relative intensity of the fast-decay (τ_1) and slow-decay (τ_2) components. As shown in **Fig. S2b**, the time constant of the fast-decay component (τ_1) decreases at high P, while that of the slow-decay component (τ_2) remains nearly unchanged. As shown in **Fig. S2c**, the relative intensity α of the fast-decay component increases significantly as a function of P. Both behaviors indicate that the fast-decay (slow-decay) component mainly comes from the Auger-assisted annihilation (radiative recombination) of IXs. The density-dependent fast-decay component on short timescales is typical of bimolecular population decay caused by Auger-assisted IX annihilation.

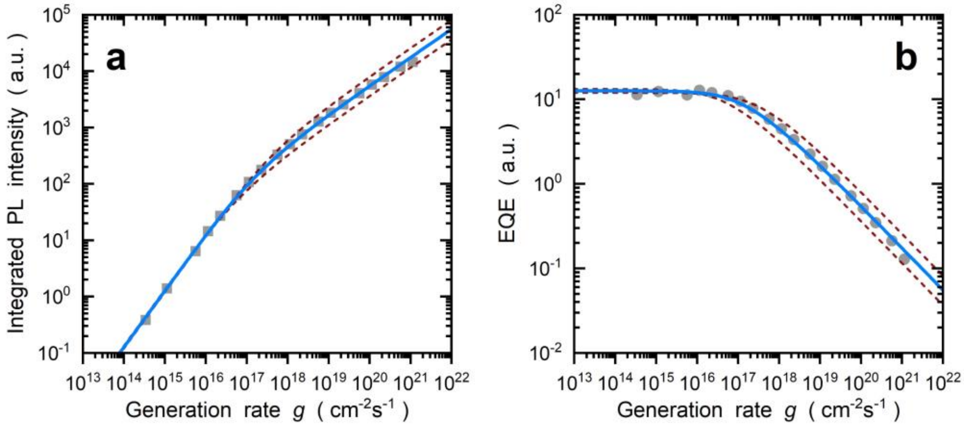

Figure S3. Determination of fitting error bars. (**a**) Integrated PL intensity and (**b**) EQE as a function of power for a WS_2/WSe_2 heterobilayer measured at T= 4K. Gray dots: PL data. Blue lines: the best fitting curves. Dash lines: the intervals of fitting deviations. Rate constants for the best fit are: $k_A = 1.3 \times 10^{-5}\ \mathrm{cm}^2\mathrm{s}^{-1}$, $k_t = 1.6 \times 10^{-3}\ \mathrm{ns}^{-1}$ and $k_R = 2.5 \times 10^5\ \mathrm{s}^{-1}$. The fitting error bar of k_A is estimated to be $0.7 \times 10^{-5}\ \mathrm{cm}^2\mathrm{s}^{-1}$.

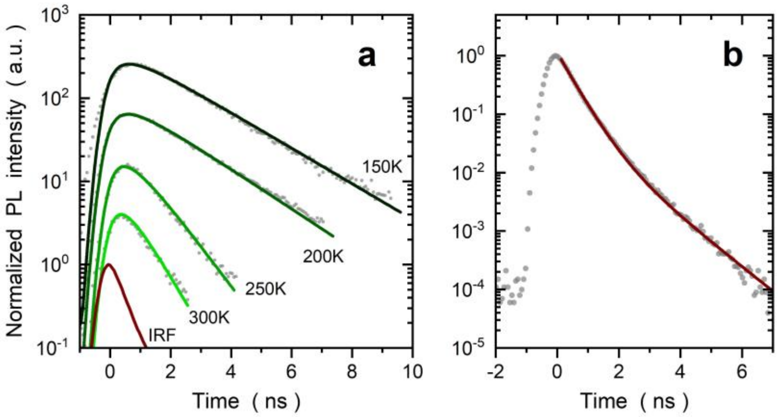

Figure S4. TRPL trace fitting at high temperatures. (**a**) TRPL traces fitted with single-exponential functions. TRPL traces have been vertically shifted for clarity. (**b**) Instrument response function (IRF) of the time-correlated single photon counting system. The dots are experimental data, and the curves are fitting results. The IRF can be fitted by: $y(t) = 0.79e^{-t/0.45} + 0.07e^{-t/1.03}$, showing an overall temporal resolution of 450 ps. TRPL traces recorded at high temperatures have been deconvoluted from the IRF. Because of the long lifetime of IXs, the IRF effect can be neglected at temperatures below 100K, as shown in **Figs. 1f** and **3c** in the main text.

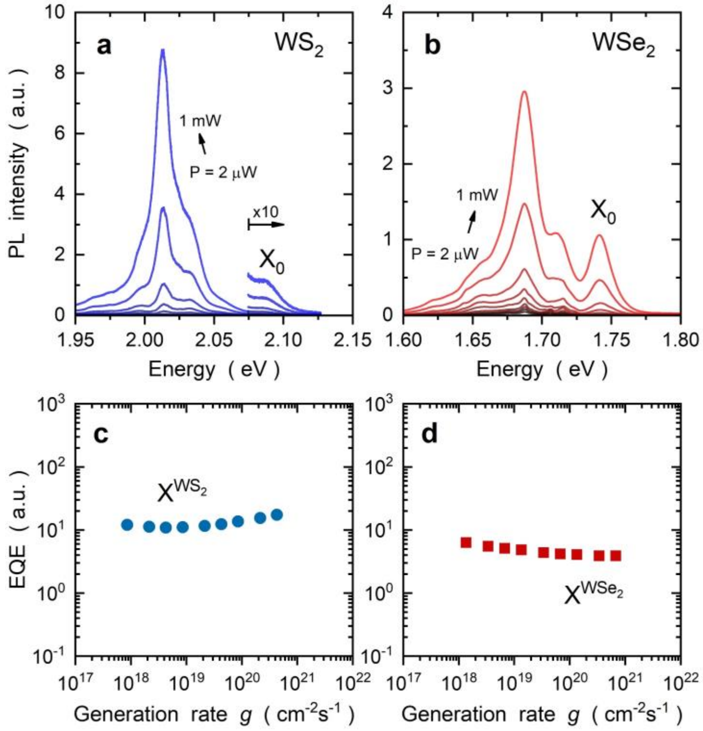

Figure S5. Power-dependent PL of monolayer WS_2 and WSe_2. (**a-b**) Power-dependent PL spectra of monolayer WS_2 (a) and monolayer WSe_2 (b) measured at *T*= 4K. It should be noted that multiple emission lines with energies lower than neutral excitons X_0 are typical of exciton complexes in tungsten-based TMDs [2-5]. The spectra in (a) with energies above 2.075 eV have been zoomed in by a factor of 10 to better visualize neutral excitons X_0. (**c-d**) Power-dependent EQE demonstrates the absence of EQE droop for both X^{WS_2} (c) and X^{WSe_2} (d). These data show that exciton-exciton annihilation (EEA) is not the primary recombination channel in this excitation range. The reason behind is likely due to the much lower exciton population (n_X) for the monolayer sample. As discussed in **Note S6**, under the same excitation conditions, the population difference between intralayer exciton and IX is estimated to be on the order of $\sim 10^3$. If we take the Auger coefficient k_A of the monolayer sample as $10^{-3} \sim 10^{-1}$ cm^{2}s^{-1} [6-12], the Auger recombination rate $k_A n_X^2$ is estimated to be $10^2 - 10^4$ lower than the heterobilayer sample. This indicates that the EQE decrease for the monolayer sample is already beyond the current maximum power.

Note S1. The power-dependent PL intensity and EQE.

The ± 0.5 exponents of I_{PL} and EQE at high power are direct features of Auger-assisted IX annihilation. Because free carriers prefer form IXs at high levels of excitation, the IX kinetic model can thus be simplified as: $dn_{IX}/dt = g - k_t n_{IX} - k_R n_{IX} - k_A n_{IX}^2$, where n_{IX} is the IX density, g is the photocarrier generation rate, k_R is the radiative recombination coefficient, and k_A is the Auger coefficient. Under steady-state excitation with a high IX density n_{IX} , we have $(k_t n_{IX} + k_R n_{IX}) \ll k_A n_{IX}^2$, which leads to $g \cong k_A n_{IX}^2$. In this regard, the power-dependent I_{PL} and EQE can be written as $I_{PL} \propto k_R n_{IX} \propto g^{0.5} \propto P^{0.5}$ and EQE $\propto I_{PL}/P \propto P^{-0.5}$, resulting in ± 0.5 exponents for I_{PL} and EQE. This rate equation analysis demonstrates that Auger-assisted IX annihilation is the dominant recombination mechanism at high power.

Note S2. Estimation of photoexcited IX density.

The photocarrier generation rate g can be calculated by: $g = FA$, where F is the photon flux density (or photon irradiance) of the incident laser, and A is the total absorbance of the WS_2/WSe_2 heterobilayer. The photon flux density F can be calculated by: $F = P/(\pi r^2 \hbar\omega)$, where P is the average power, $\hbar\omega = 2.33$ eV is the photon energy and πr^2 is the spot size of the focused 532-nm laser. The overall absorbance of the WS_2/WSe_2 heterobilayer is estimated to be $A = 9.7\%$ utilizing the transfer matrix method to solve electric field distributions in the multilayer structure. The model takes into account the absorbance of TMD layers as well as the reflection/transmission losses of the multilayer structure. The transmission loss of the vacuum chamber window and the objective lens is also considered. Under continuous-wave laser excitation with an average power $P = 1\ \mu W$, the generation rate is estimated to be $g = 1.13 \times 10^{18}\ \mathrm{cm^{-2}s^{-1}}$. At the slope conversion point with $P = 200$ nW, we estimate a steady-state IX density of $8.4 \times 10^{10}\ \mathrm{cm^{-2}}$ at $T = 4$K, which is significantly lower than the Mott transition point reported for TMD hetero-bilayers [13,14].

Note S3. Calculation of IX annihilation rate using Fermi's golden rule.

As shown in **Fig. 2e** in the main text, an Auger-assisted IX annihilation event is directly linked to one IX being recombined nonradiatively with energy absorbed by another IX to its excited state concurrently. In this case, the IX annihilation rate can be calculated using Fermi's golden rule between two interacting dipoles. The transition rate from an initial state $|\psi_i\rangle$ with total energy E_i to a final state $|\psi_f\rangle$ with total energy E_f is given by: $\Gamma_{i\to f} = \frac{2\pi}{\hbar}\left|\langle\psi_f|\hat{V}|\psi_i\rangle\right|^2\delta(E_f - E_i)$. The initial state is two IXs with energy E_{IX}, while the final state is one IX with energy $2E_{IX}$, resulting in $E_f - E_i = 0$. The dipole-dipole interaction potential can be approximated as $\mathrm{V} \propto \mu_D\mu_A/R^3$, where μ_D (μ_A) is the transition dipole moment of the energy "donor" ("acceptor") IX and R is the inter-excitonic distance. By assuming that the wavefunction can be separated as $|\psi_i\rangle = |\psi_i^D\rangle|\psi_i^A\rangle$ and $|\psi_f\rangle = |\psi_0^D\rangle|\psi_f^A\rangle$, one can thus obtain the Auger-assisted IX annihilation rate as: $\Gamma_{i\to f} = \frac{2\pi}{\hbar}\left|\langle\psi_f|\hat{V}|\psi_i\rangle\right|^2 \propto \left|\langle\psi_0^D|\mu_D|\psi_i^D\rangle\right|^2\left|\langle\psi_f^A|\mu_A|\psi_i^A\rangle\right|^2$. It should be noted that the first matrix element $\left|\langle\psi_0^D|\mu_D|\psi_i^D\rangle\right|^2$ is proportional to the energy donor IX's radiative recombination rate, whereas the second matrix element $\left|\langle\psi_f^A|\mu_A|\psi_i^A\rangle\right|^2$ is proportional to the energy acceptor IX's absorption rate to the high-energy continuum state. Therefore, a weak oscillator strength (a long radiative lifetime) leads to a low Auger annihilation coefficient. This is the reason behind the ultralow Auger coefficient of IXs in TMD heterobilayers compared to excitons in TMD monolayers.

Note S4. Fitting of temperature-dependent IX kinetics.

The proposed kinetic model accurately reproduces the entire temperature-dependent behavior of PL intensity and EQE. Two separate fitting sets are employed to examine the roles of k_A and k_t. In the first fitting set shown in **Fig. S6**, we only change k_t while keeping the other coefficients fixed. This fitting set already gives an adequate fit to all experimental data (**Figs. S6a-b**) and captures the effects of thermally activated k_t (**Fig. S6c**). The fixed parameters are $k_R = 2.5 \times 10^5\ \mathrm{s}^{-1}$, and $k_A = 1.3 \times 10^{-5}\ \mathrm{cm}^2\mathrm{s}^{-1}$. Nonetheless, the second fitting set provided in the main text obviously fits all experimental data better (see **Fig. 3** in the main text). It is worth noting that both fitting sets demonstrate that the Auger coefficient is on the order of $10^{-5}\ \mathrm{cm}^2\mathrm{s}^{-1}$. On

the other hand, we determine an efficient $k_t = 13.4\ \mathrm{ns}^{-1}$ at room temperature, which is quite similar to the previously reported value [15]. As a result, determining k_A for IXs at $T \geq 150\mathrm{K}$ becomes problematic. For example, the time constant of IX annihilation is 20 ns for an IX density of around $10^{12}\ \mathrm{cm}^{-2}$. At $T \geq 150\mathrm{K}$, however, the average nonradiative time k_t^{-1} is already less than 1 ns, which can cause a significant inaccuracy in determining k_A. As a result, our experimental findings show that nonradiative recombination must be considered when analyzing IX kinetics at high temperatures, especially with such a low Auger-assisted IX annihilation rate.

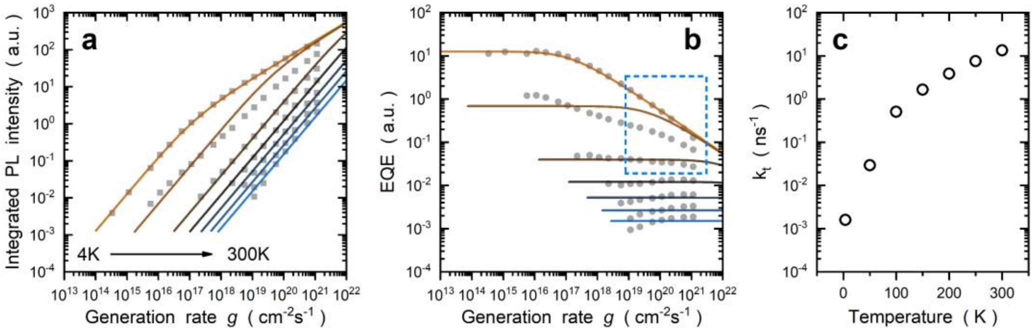

Figure S6. The influence of temperature on interlayer exciton kinetics. (**a-b**) Density dependence of integrated PL intensity (a) and EQE (b) measured at elevated temperatures. The dots are experimental data, and the curves are fitting results. (**c**) Temperature dependence of k_t. In this fitting set, we only change k_t while keeping the other coefficients fixed. This fitting set already gives an adequate fit to all experimental data and captures the effects of thermally activated k_t. The most inaccurate fits are found at *T*= 50K and 100K, as shown by the blue dashed area. It should be noted that, while this fitting set provides an adequate fit, the second fitting set provided in the main text obviously gives a better fit to all experimental data (see **Fig. 3** in the main text).

On the other hand, we notice a trend of super-linear PL intensity and EQE enhancement with P at high temperatures. The reason behind might involve competition between hot-carrier recombination and IX formation. We propose here a potential correction term for use at high temperatures: $dn_c/dt = g - k_t n_c - k_b n_c^2$; $dn_{IX}/dt = k_b n_c^2 - k_t n_{IX} - k_R n_{IX} - k_A n_{IX}^2$, where n_c is the hot-carrier density and the term $k_b n_c^2$ represents IX formation through Coulomb binding

of free carriers. As shown in **Fig. S7**, these equations fit the high-temperature data well, as indicated by the black arrow. However, further studies are needed to verify detailed processes, including interlayer carrier transfer and thermalization processes.

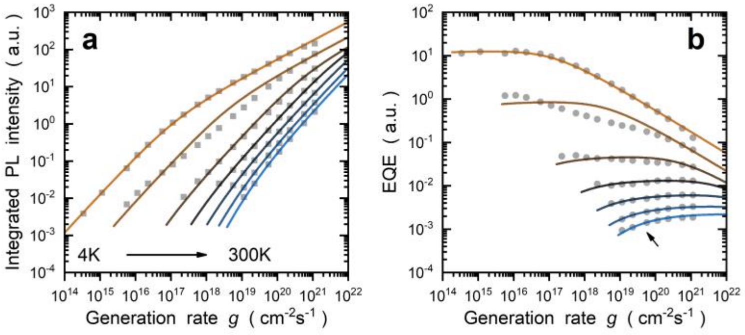

Figure S7. The influence of temperature on interlayer exciton kinetics. (**a-b**) Density dependence of integrated PL intensity (a) and EQE (b) measured at elevated temperatures. The dots are experimental data, and the curves are fitting results.

Note S5. Auger recombination of delocalized IXs in WS_2/WSe_2 heterobilayer.

We note that phonon-assisted recombination/relaxation of IXs also plays an important role in TMD moiré heterobilayers. However, since the phonon-assisted process is a low-order process, it cannot explain bimolecular recombination at a rate ($k_A n_{IX}^2$) that depends quadratically on IX density, as shown by the characteristic EQE droop and density-dependent TRPL decay. We realize that, while this Auger recombination occurs under high-power/low-temperature conditions, the phonon-assisted process is reflected in the nonradiative recombination rate $k_t n_{IX}$, which dominates IX recombination at high temperatures. Additionally, we realize that the type of IXs may also play a role. Specifically, our results show Auger recombination of delocalized IXs, which behaves differently from moiré-trapped IXs. In the following, we compare their spectral characteristics and demonstrate delocalized IXs using diffusion measurements.

Several important papers have demonstrated the dynamics of moiré-trapped IXs in $MoSe_2/WSe_2$ heterobilayers [16-20]. Crucially, it is shown that IX recombination/relaxation is governed by phonon-assisted processes involving different moiré potential minima and transitions between bright and dark states [17-20]. The PL emission of moiré-trapped IXs usually exhibits: (**1**) a narrow linewidth (~1 meV) at low power; (**2**) a power-broadening linewidth; and (**3**) a rapid power saturation due to state-filling effect. However, IX emissions in WS_2/WSe_2 heterobilayers exhibit different PL features, including: (**1**) a broad linewidth of 48 meV at low power (**Fig. S8a**); (**2**) a power-insensitive linewidth (**Fig. S8a**); and (**3**) PL efficiency droop ($\mathrm{EQE} \propto P^{-0.5}$) spanning nearly 4 orders of magnitude power range (**Fig. S8b**). Combining density-dependent PL with model fitting, we therefore conclude that it is nonradiative bimolecular recombination caused by Auger recombination between two IXs.

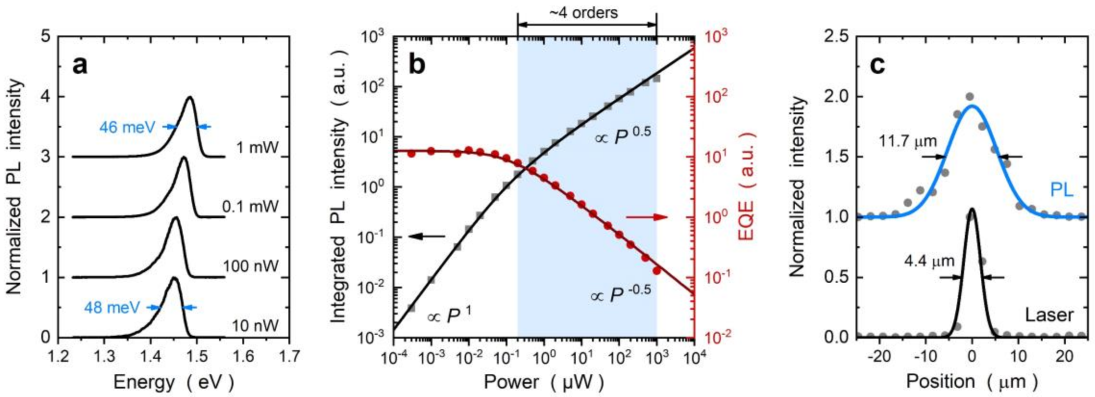

Figure S8. Interlayer excitons in the WS_2/WSe_2 heterobilayer. (**a**) Normalized PL spectra at $T = 4\mathrm{K}$. As laser power increases, IX emission exhibits a nearly constant linewidth. (**b**) The integrated PL intensity and EQE of IX emission as a function of power. Note that PL efficiency droop spans nearly 4 orders of magnitude power range (blue region). (**c**) Spatially-resolved PL and laser images with $\mathrm{P} = 10$ nW, where the data (dots) are fitted by Gaussian functions (lines). IX diffusion is evidenced by the wider PL profile beyond the laser spot.

Microscopically, our results are also consistent with delocalized excitons that can diffuse over a certain distance [6,10,21-24]. In the WS_2/WSe_2 heterobilayer, Auger recombination occurs when the IX density exceeds $8.4 \times 10^{10}\ \mathrm{cm}^{-2}$, which corresponds to an average IX separation of 37 nm. This distance is much larger than the moiré periodicity (~8 nm) and the spatial extent of an IX, indicating that diffusion must precede Auger recombination [6,10]. As shown in **Fig. S8c**, spatially-resolved PL images reveal IX diffusion in the WS_2/WSe_2 heterobilayer, demonstrating that IXs are delocalized from the moiré potential. The distinct IX dynamics observed in the WS_2/WSe_2 heterobilayer indicate a rich moiré exciton phenomenon in TMD heterobilayers. We also realize that the difference between $MoSe_2/WSe_2$ and WS_2/WSe_2 heterobilayers may be related to moiré periodicity and specific excitation/material conditions [21-24], resulting in moiré-trapped IX phase for the former while delocalized IX phase for the latter.

Note S6. The negligible effect of intralayer excitons on IX recombination.

Here, we have included additional TRPL data to clarify the interaction between exciton and IX. Below, we show the negligible effect of intralayer excitons on IX recombination, which is concluded from their significant difference in exciton lifetime and population on the order of $\sim 10^3$. This ensures that IX-IX interactions are responsible for nonradiative bimolecular recombination. **Figure S9** shows the TRPL traces of WS_2 excitons, WSe_2 excitons, and IXs in the WS_2/WSe_2 heterobilayer measured at $T = 4$K. The PL spectrum reveals various intralayer exciton complexes including: neutral exciton X^0, charged exciton T, and other exciton complexes (**Fig. S9a**). It is observed that intralayer excitons exhibit much faster decays compared to IXs (**Figs. S9b-d**). The main decay lifetimes of WS_2 excitons and WSe_2 excitons are in the range of $0.2 - 0.5$ ns (**Figs. S9c-d** and **Table S1**), which are significantly shorter than the IX lifetime (> 450 ns) and the time constant of IX-IX annihilation (~20 ns). This large lifetime difference therefore results in the negligible effect of intralayer excitons on IX recombination in TRPL measurements. On the other hand, in PL measurements using continuous-wave laser excitation, the lifetime difference translates into the difference in steady-state exciton population, i.e., yielding a huge population difference on the order of $\sim 10^3$. In this context, our results demonstrate that IX recombination is primarily affected by IX-IX interactions.

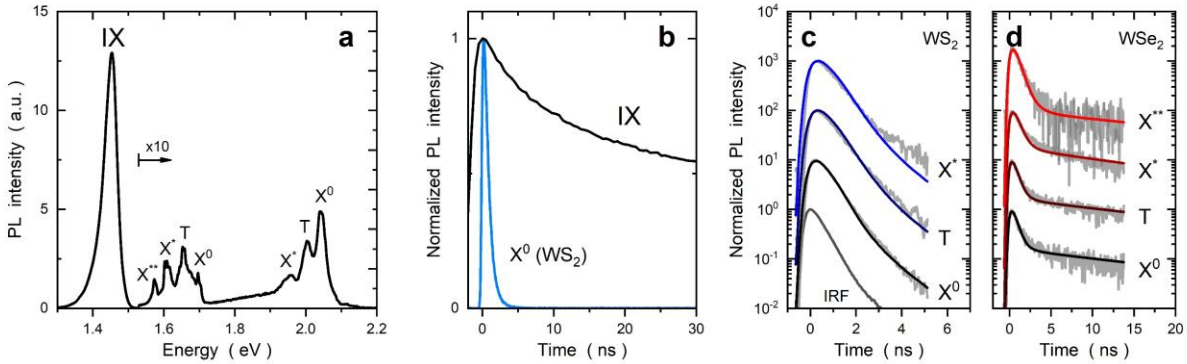

Figure S9. TRPL of intralayer excitons in the WS_2/WSe_2 heterobilayer. (**a**) The PL spectrum at *T*= 4K shows emissions from WS_2 excitons, WSe_2 excitons, and IXs. The spectrum with energies above 1.53 eV has been zoomed in by a factor of 10. (**b**) The TRPL traces of WS_2 neutral excitons and IXs in the WS_2/WSe_2 heterobilayer, showing a much faster decay of WS_2 neutral excitons. TRPL traces of WS_2 excitons (**c**) and WSe_2 excitons (**d**), in which TRPL traces have been shifted vertically for clarity. TRPL traces have been deconvoluted from the IRF and fitted with single-exponential (double-exponential) functions for WS_2 excitons (WSe_2 excitons). The gray lines are experimental data, and the colored curves are fitting results.

**(Unit: ns)**	τ_1	τ_2
X^0 (WS_2)	0.27	n/a
T (WS_2)	0.39	n/a
X^* (WS_2)	0.40	n/a
X^0 (WSe_2)	0.29	22.20
T (WSe_2)	0.31	22.29
X^* (WSe_2)	0.48	18.57
X^{**} (WSe_2)	0.52	25.00

Table S1. Lifetimes of WS_2 excitons and WSe_2 excitons in the WS_2/WSe_2 heterobilayer. Note that τ_1 may not be determined accurately since the overall time resolution of the TRPL system is ~0.45 ns.

Finally, the interaction between IX and intralayer exciton in moiré superlattice is an important topic to explore. Following the above discussion, we realize that studying such interaction requires probing exciton dynamics on sub-nanosecond time scales, which is not feasible in current TRPL experiments due to limited time resolution (~0.45 ns). We anticipate that more advanced investigations, such as pump-probe measurements, will be needed to study these exciton interactions.

References:

1. C. Jin, E. C. Regan, A. Yan, M. Iqbal Bakti Utama, D. Wang, S. Zhao, Y. Qin, S. Yang, Z. Zheng, S. Shi, K. Watanabe, T. Taniguchi, S. Tongay, A. Zettl, and F. Wang, Observation of moiré excitons in WSe_2/WS_2 heterostructure superlattices. *Nature* **567**, 76-80 (2019).
2. M. Paur, A. J. Molina-Mendoza, R. Bratschitsch, K. Watanabe, T. Taniguchi, and T. Mueller, Electroluminescence from multi-particle exciton complexes in transition metal dichalcogenide semiconductors. *Nat. Commun.* **10**, 1709 (2019).
3. Z. Li, T. Wang, C. Jin, Z. Lu, Z. Lian, Y. Meng, M. Blei, S. Gao, T. Taniguchi, K. Watanabe, T. Ren, S. Tongay, L. Yang, D. Smirnov, T. Cao, and S. F. Shi, Emerging photoluminescence from the dark-exciton phonon replica in monolayer WSe_2. *Nat. Commun.* **10**, 2469 (2019).
4. Y. Tang, K. F. Mak, and J. Shan, Long valley lifetime of dark excitons in single-layer WSe_2. *Nat. Commun.* **10**, 4047 (2019).
5. M. He, P. Rivera, D. Van Tuan, N. P. Wilson, M. Yang, T. Taniguchi, K. Watanabe, J. Yan, D. G. Mandrus, H. Yu, H. Dery, W. Yao, and X. Xu, Valley phonons and exciton complexes in a monolayer semiconductor. *Nat. Commun.* **11**, 618 (2020).
6. L. Yuan and L. Huang, Exciton dynamics and annihilation in WS_2 2D semiconductors. *Nanoscale* **7**, 7402-7408 (2015).
7. Y. Hoshi, T. Kuroda, M. Okada, R. Moriya, S. Masubuchi, K. Watanabe, T. Taniguchi, R. Kitaura, and T. Machida, Suppression of exciton-exciton annihilation in tungsten disulfide monolayers encapsulated by hexagonal boron nitrides. *Phys. Rev. B* **95**, 241403 (2017).
8. Y. Lee, J. D. S. Forte, A. Chaves, A. Kumar, T. T. Tran, Y. Kim, S. Roy, T. Taniguchi, K. Watanabe, A. Chernikov, J. I. Jang, T. Low, and J. Kim, Boosting quantum yields in two-dimensional semiconductors via proximal metal plates. *Nat. Commun.* **12**, 7095 (2021).
9. Y. Lee, T. T. Tran, Y. Kim, S. Roy, T. Taniguchi, K. Watanabe, J. I. Jang, and J. Kim, Enhanced radiative exciton recombination in monolayer WS_2 on the hBN substrate competing with nonradiative exciton-exciton annihilation. *ACS Photonics* **9**, 873-879 (2022).
10. S. Mouri, Y. Miyauchi, M. Toh, W. Zhao, G. Eda, and K. Matsuda, Nonlinear photoluminescence in atomically thin layered WSe_2 arising from diffusion-assisted exciton-exciton annihilation. *Phys. Rev. B* **90**, 155449 (2014).
11. D. F. Cordovilla Leon, Z. Li, S. W. Jang, and P. B. Deotare, Hot exciton transport in WSe_2 monolayers. *Phys. Rev. B* **100**, 241401 (2019).
12. D. Erkensten, S. Brem, K. Wagner, R. Gillen, R. Perea-Causín, J. D. Ziegler, T. Taniguchi, K. Watanabe, J. Maultzsch, A. Chernikov, and E. Malic, Dark exciton-exciton annihilation in monolayer WSe_2. *Phys. Rev. B* **104**, L241406 (2021).
13. J. Wang, J. Ardelean, Y. Bai, A. Steinhoff, M. Florian, F. Jahnke, X. Xu, M. Kira, J. Hone, and X.-Y. Zhu, Optical generation of high carrier densities in 2D semiconductor heterobilayers. *Sci. Adv.* **5**, eaax0145 (2019).
14. J. Wang, Q. Shi, E. M. Shih, L. Zhou, W. Wu, Y. Bai, D. Rhodes, K. Barmak, J. Hone, C. R. Dean, and X.-Y. Zhu, Diffusivity reveals three distinct phases of interlayer excitons in

$MoSe_2/WSe_2$ heterobilayers. *Phys. Rev. Lett.* **126**, 106804 (2021).

15. D. Sun, Y. Rao, G. A. Reider, G. Chen, Y. You, L. Brézin, A. R. Harutyunyan, and T. F. Heinz, Observation of rapid exciton-exciton annihilation in monolayer molybdenum disulfide. *Nano Lett.* **14**, 5625-5629 (2014).
16. M. Brotons-Gisbert, H. Baek, A. Campbell, K. Watanabe, T. Taniguchi, and B. D. Gerardot, Moiré-trapped interlayer trions in a charge-tunable $WSe_2/MoSe_2$ heterobilayer. *Phys. Rev. X* **11**, 031033 (2021).
17. K. Shinokita, Y. Miyauchi, K. Watanabe, T. Taniguchi, and K. Matsuda, Resonant coupling of a moiré exciton to a phonon in a $WSe_2/MoSe_2$ heterobilayer. *Nano Lett.* **21**, 5938-5944 (2021).
18. K. Shinokita, K. Watanabe, T. Taniguchi, and K. Matsuda, Valley relaxation of the moiré excitons in a $WSe_2/MoSe_2$ heterobilayer. *ACS Nano* **16**, 16862-16868 (2022).
19. H. Kim, D. Dong, Y. Okamura, K. Shinokita, K. Watanabe, T. Taniguchi, and K. Matsuda, Dynamics of moiré trion and its valley polarization in a microfabricated $WSe_2/MoSe_2$ heterobilayer. *ACS Nano* **17**, 13715-13723 (2023).
20. H. Kim, K. Aino, K. Shinokita, W. Zhang, K.Watanabe, T. Taniguchi, and K. Matsuda, Dynamics of moiré exciton in a twisted $MoSe_2/WSe_2$ heterobilayer. *Adv. Optical Mater.* **11**, 2300146 (2023).
21. S. Brem, C. Linderälv, P. Erhart, and E. Malic, Tunable phases of moiré excitons in van der Waals heterostructures. *Nano Lett.* **20**, 8534-8540 (2020).
22. J. Choi, W.-T. Hsu, L.-S. Lu, L. Sun, H.-Y. Cheng, M.-H. Lee, J. Quan, K. Tran, C.-Y. Wang, M. Staab, K. Jones, T. Taniguchi, K. Watanabe, M.-W. Chu, S. Gwo, S. Kim, C.-K. Shih, X. Li, and W.-H. Chang, Moiré potential impedes interlayer exciton diffusion in van der Waals heterostructures. *Sci. Adv.* **6**, eaba8866 (2020).
23. W. Knorr, S. Brem, G. Meneghini, and E. Malic, Exciton transport in a moiré potential: From hopping to dispersive regime. *Phys. Rev. Mater.* **6**, 124002 (2022).
24. S. Brem and E. Malic, Bosonic delocalization of dipolar moiré excitons. *Nano Lett.* **23**, 4627-4633 (2023).
